# Photothermal Fibrous Chitosan/Polydopamine Sponge for Intraoperative Hemostasis and Prevention of Tumor Recurrence in Hepatocellular Carcinoma Resection

**DOI:** 10.1002/advs.202304053

**Published:** 2023-11-29

**Authors:** Lanxin Mu, Luhe Qi, Haitao Long, Jing Huang, Zibiao Zhong, Xiaowen Shi, Chaoji Chen, Qifa Ye

**Affiliations:** ^1^ National Quality Control Center for Donated Organ Procurement Hubei Key Laboratory of Medical Technology on Transplantation Hubei Clinical Research Center for Natural Polymer Biological Liver Hubei Engineering Center of Natural Polymer‐based Medical Materials, Zhongnan Hospital of Wuhan University Institute of Hepatobiliary Diseases of Wuhan University, Transplant Center of Wuhan University Wuhan 430071 China; ^2^ School of Resource and Environmental Science Hubei International Scientific and Technological Cooperation Base of Sustainable Resource and Energy Hubei Engineering Center of Natural Polymers‐Based Medical Materials Hubei Biomass‐Resource Chemistry and Environmental Biotechnology Key Laboratory Wuhan University Wuhan 430079 China

**Keywords:** hemostasis, immunotherapy, lenvatinib, liver cancer recurrence, photothermal therapy

## Abstract

Hepatectomy, a surgical procedure for liver cancer, is often plagued by high recurrence rates worldwide. The recurrence of liver cancer is primarily attributed to microlesions in the liver, changes in the immune microenvironment, and circulating tumor cells in the bloodstream. To address this issue, a novel intervention method that combines intraoperative hemostasis with mild photothermal therapy is proposed, which has the potential to ablate microlesions and improve the immune microenvironment simultaneously. Specifically, the integrated strategy is realized based on the fibrous chitosan/polydopamine sponge (CPDS), which is constructed from shearing‐flow‐induced oriented hybrid chitosan fibers and subsequent self‐assembly of polydopamine. The CPDS demonstrates high elasticity, excellent water absorption, and photothermal conversion performance. The results confirm the efficient hemostatic properties of the fibrous CPDS in various bleeding models. Notably, in subcutaneous and orthotopic postoperative recurrence and metastasis models of hepatocellular carcinoma, the fibrous CPDS significantly inhibits local tumor recurrence and distant metastasis. Moreover, the combination with lenvatinib can substantially enhance the antitumor effect. This comprehensive treatment strategy offers new insights into hepatectomy of liver cancer, representing a promising approach for clinical management.

## Graphical abstract

A photothermal fibrous chitosan/polydopamine sponge, constructed from shearing‐flow‐induced oriented hybrid chitosan fibers, is presented to enhance hepatectomy outcomes by combining intraoperative hemostasis with mild photothermal therapy. This strategy efficiently addresses bleeding, inhibits tumor recurrence and metastasis in various models, particularly when combined with lenvatinib, offering significant promise for improving liver cancer surgery.

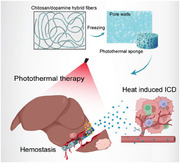

## 1 Introduction

Liver cancer is a prevalent malignant disease that ranks fifth globally, responsible for 9.1% of all cancer‐related fatalities.^[^ ^1^ ^]^ At present, surgical resection remains the preferred and most efficacious treatment method for liver cancer.^[^ ^2^ ^]^ Nevertheless, the five‐year recurrence rate following liver resection can reach as high as 50–70%, indicating that mitigating postoperative recurrence is pivotal to enhancing the overall curative efficacy of liver cancer.^[^ ^3^ ^]^ Unfortunately, there is no universally acknowledged postoperative adjuvant therapy for liver cancer, due to the three major factors of hepatocellular carcinoma (HCC) recurrence: 1) following circulating tumor cells in the blood; 2) microlesions in the liver, and 3) changes of the immune microenvironment.^[^ ^4^, ^5^ ^]^ Growing evidence suggests that the process of tumor resection in liver cancer patients can inadvertently lead to the dissemination of tumor cells into the bloodstream due to inevitable intraoperative hemorrhage.^[^ ^4^ ^]^ This phenomenon subsequently elevates the levels of circulating tumor cells (CTCs), thereby increasing the risk of both local recurrence and distant metastasis.^[^ ^4^ ^]^ Furthermore, microlesions that are not removed can develop after surgery, due to enhanced regenerative capacity after hepatectomy and altered immune microenvironment.^[^ ^5^, ^6^ ^]^

Recently, local therapeutic materials with hemostatic properties have been developed for liver cancer resection surgery.^[^ ^7^ ^]^ While sponges seem to be an effective form of hemostatic agent due to their excellent bloodsucking ability,^[^ ^8^, ^9^, ^10^, ^11^ ^]^ most studies suppress tumor recurrence by adding drugs to the hemostatic agent. Nonetheless, these localized therapeutic approaches lack the ability to elicit systemic immune responses comparable to thermotherapy. Currently, there is no safer local treatment material used in liver resection that can simultaneously absorb blood, ablate microlesions, and activate immunity. Thus, a combined strategy of hemostasis and photothermal therapy for the prevention of recurrence following liver cancer resection is yet to be established.

**Figure 1:**
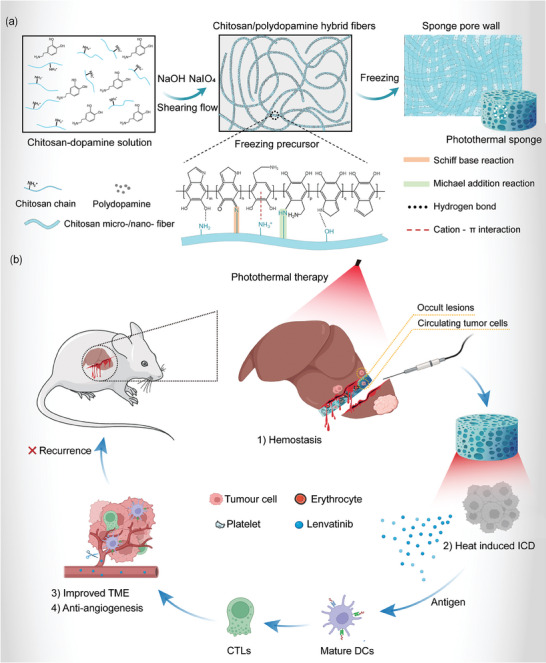
a) Schematic diagram of CPDS synthesis. b) Schematic illustration of CPDS‐mediated immunotherapy inhibits tumor recurrence. 1) Effective hemostasis can absorb circulating tumor cells and prevent cancer from spreading. 2) PTT ablates occult lesions and induced ICD. 3) The ICD of occult lesions promotes the maturation of dendritic cells (DCs) and the increase of cytotoxic T lymphocytes (CTLs), which finally improves the tumor immune microenvironment (TME). 4) Combined use of lenvatinib can inhibit angiogenesis and block tumor nutrition. All the above mechanisms work together to inhibit the recurrence of hepatocellular tumors.

Herein, we created a photothermal fibrous chitosan/polydopamine sponge (CPDS) for dual benefits with bolstered hemostatic efficacy while concurrently enhancing antitumor activity. The shearing‐flow‐induced oriented hybrid chitosan/polydopamine fibers (**Figure** **1**a) endow the as‐prepared sponge with exceptional mechanical properties, remarkable blood‐absorbing properties, and enhanced hemostatic efficacy thereby reducing the potential for tumor cell extravasation (Figure 1b). Meanwhile, polydopamine, as the promising candidate for photothermal therapy (PTT) material with high biodegradability and low potential toxicity, exhibited efficient light‐to‐heat conversion with satisfactory antitumor properties.^[^ ^12^ ^]^ Additionally, by leveraging thermal ablation, the fibrous CPDS not only reduces local tumor burden but also harnesses the power of immunogenic cell death (ICD) to promote the release of tumor‐associated antigens (TAAs),^[^ ^13^ ^]^ which elicits a systemic immune response and promotes the regression of tumors located elsewhere in the body (Figure 1b). Furthermore, we have explored whether a combination of a photothermal sponge and Lenvatinib (LT), a first‐line drug for liver cancer, has a synergistic effect and we have drawn positive conclusions (Figure 1b). Our work demonstrates an effective and sustainable strategy for liver cancer treatment that can simultaneously mitigate intraoperative bleeding and minimize the likelihood of postoperative recurrence by multifunctional use of renewable biomass.

## 2 Results and Discussion

### 2.1 Structure and Properties of the CPDS

The chitosandopamine solution geled and be oxidized respectively by adding NaOH solution and NaIO_4_, accompanied by high‐speed shearing with a homogenizer. The mixture self‐assembled into composite microfibers with high aspect ratio under the shear‐flow, which is similar to the results in our previous works.^[^ ^14^, ^15^ ^]^ The polydopamine nanoparticles embedded in the fibers make the fibers rough as shown in the transmission electron microscope (Figure S1, Supporting Information). Subsequently, the as‐prepared precursor was frozen and thawed to form the macroporous networks without extra crosslinker. Finally, the CPDS was obtained by ethanol solvent exchange and ambient drying.

**Figure 2:**
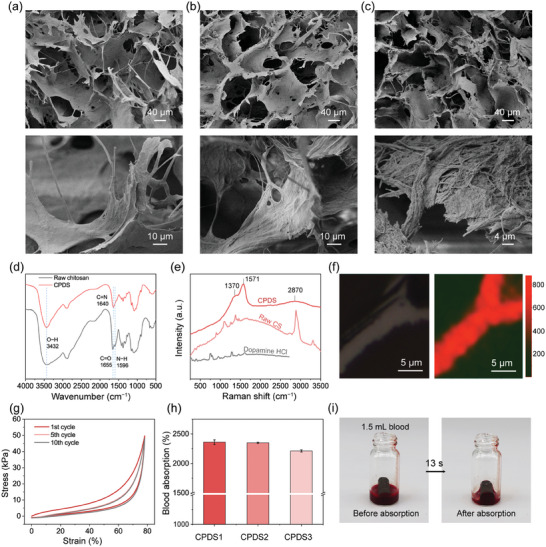
Structure and properties of the fibrous CPDS. The SEM images of a) CPDS1, b) CPDS2, and c) CPDS3 respectively. d) The FT‐IR spectra of raw chitosan powder and CPDS. e) The 1D Raman spectra of raw chitosan powder, dopamine HCl, and CPDS. f) The 2D images from the aromatic C─C vibrations (1500–1590 cm^−1^) within the CPDS1 were derived from a multivariate curve resolution model with OMNIC software. g) The cycle compression stress–strain curves of the CPDS2. h) The blood absorption capability of the CPDS. i) The photographs of the blood absorption capability of CPDS2: absorbing 1.5 mL of blood in ≈13 s.

An interconnected porous structure and abundant fibers on the pore walls were exhibited on the resulting CPDSs. More fibers in the sponge network were observed with the increase of polydopamine (PDA) , and the diameter of the fibers changed towards nano size, as shown in the high magnification of the scanning electron microscope (SEM) (**Figure** **2**a–c). This result confirmed that the shearing flow induces the mixture of chitosan and PDA to form long fibers instead of smashing the mixture. The rough topography of CPDS facilitates the adsorption and immobilization of blood cells, fibrin, and free tumor cells that may exist in the blood.

The Fourier transform infrared spectroscopy (FTIR) spectra showed that, compared with the chitosan raw material, the absorption peak of the CPDS1 was weakened at 1596 cm^−1^ from the flexural vibration of amino groups, meanwhile, a new peak appeared at 1640 cm^−1^ (Figure 2d). These results implied the chemical crosslinking between the oxidized dopamine and the amino groups of the chitosan .^[^ ^10^ ^]^ The Raman spectrum of the CPDS exhibited completely different characteristics compared to those of the raw chitosan and dopamine HCl: Two weak bands were observed at 1571 and 1370 cm^−1^, which refer to the C─C vibrations of the aromatic units (Figure 2e). Meanwhile, from the 2D Raman analysis of the CPDS, red regions representing the C─C vibrations of the aromatic units from polydopamine (1500–1590 cm^−1^) were revealed. It indicated that the polydopamine was broadly and uniformly distributed in the CPDS,^[^ ^16^ ^]^ which further endowed the sponges with excellent photothermal effects and enhanced hemostatic performance (Figure 2f). In addition, abundant interactions existed between chitosan and polydopamine were reported, including covalent cross‐linking (such as Schiff base reaction,^[^ ^17^ ^]^ Michael addition reaction^[^ ^18^ ^]^), and non‐covalent interactions (such as hydrogen bond , cation─π interaction,^[^ ^18^ ^]^ etc.), which made the chitosan and polydopamine system form a stable 3D network.

High porosity (95%–98%) (Figure S2, Supporting Information), hydrophily (Figure S3, Supporting Information), and high elasticity were exhibited by the CPDS, making it a promising hemostatic sponge that could rapidly absorb blood and conform to wound cavities. The CPDS also exhibit high compressibility, excellent mechanical strength (≈29.1–49.7 kPa), and high elasticity and mechanical stability with at least ten times stable compression cycles (Figure 2g; Figure S4, Supporting Information). These propertieswere attributed to the tough network structure formed by non‐covalent and covalent crosslinked chitosan/polydopamine fibers with high aspect ratios. The CPDS sponges presented a 19.7–27.2 times water absorption ratio. It could absorb blood or body fluids rapidly within tens of seconds, with a blood absorption ratio of 22.10−23.60 times (Figure 2h,i; Figure S5, Supporting Information). These properties benefit from the unique pH‐induced gelation step combined with mechanical shearing,^[^ ^14^ ^]^ which also offers flexibility in shaping the sponge according to specific requirements (Figure S6, Supporting Information). These results suggest that the CPDS is potential for clinical applications in cooperative hemostasis and antitumor therapy.

### 2.2 Hemostatic Performance

**Figure 3:**
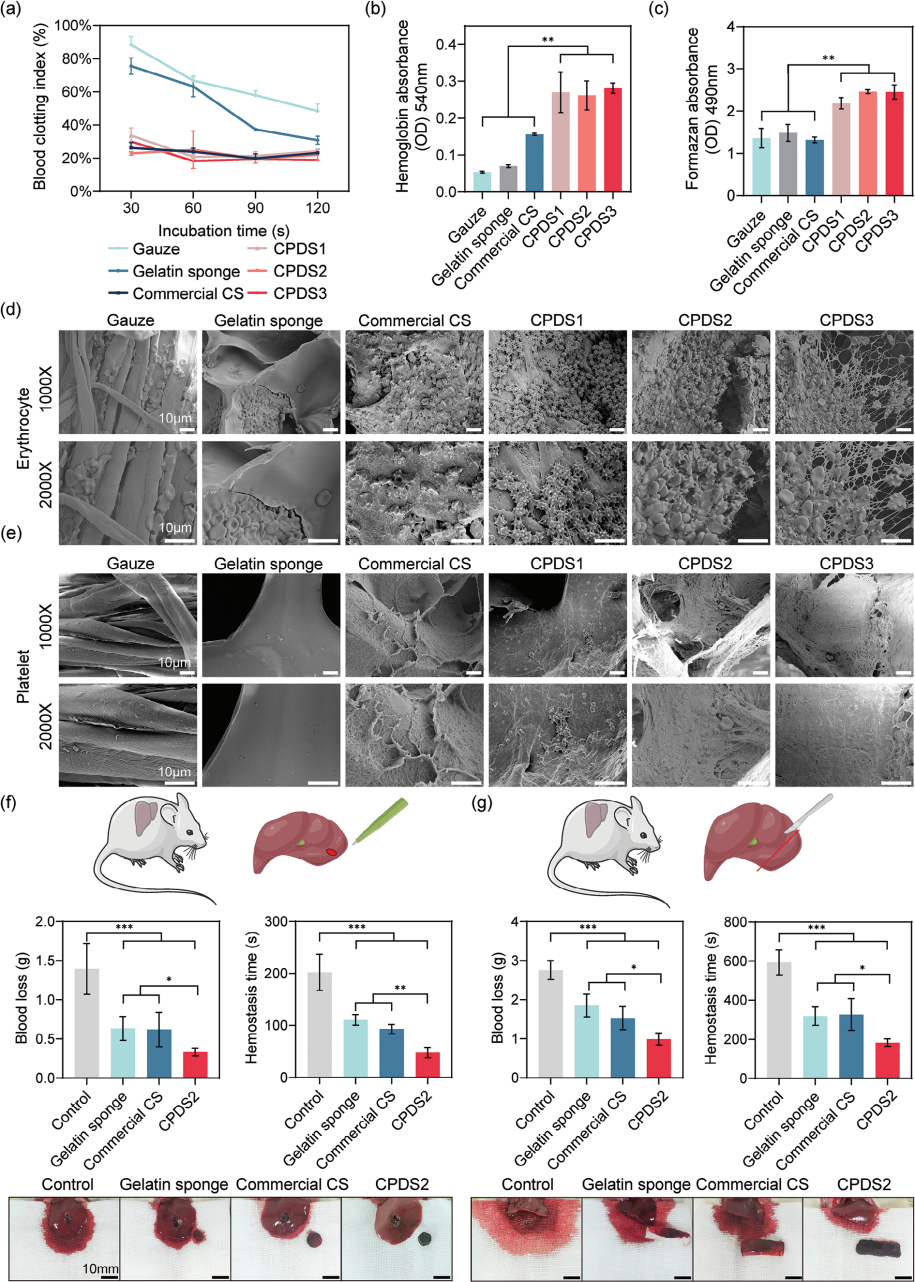
CPDSs have excellent hemostatic properties and can effectively stop bleeding in animal models of hepatic noncompressible hemorrhage. ^* * *^ *p* < 0.001, ^* *^ *p* < 0.01, ^*^ *p* < 0.05. a) Dynamic whole blood clotting assay of different hemostatic materials. b) Evaluate the amount of red blood cells and c) platelets adhered to different materials after exposure to blood. d) SEM showing adherent red blood cells and e) platelets. Scale bar: 10 µm. f) Schematic diagram, blood loss, hemostatic time, and photographs of standard round defect liver hemorrhage model (*n* = 5). g) Schematic diagram, blood loss, hemostatic time, and photographs of partial hepatectomy wound bleeding model (*n* = 5). Scale bar: 10 mm.

The hemostatic effectiveness of the CPDS was tested through a dynamic whole blood clotting assay. The study showed that CPDS exhibited rapid hemostasis, outperforming gelatin sponge and gauze at 30, 60, and 90 s (*p* < 0.05) (**Figure** **3**a). After 120 s, the hemostatic effects of other materials gradually approached CPDS, except for the gauze. The hemostatic effect of commercial chitosan was similar to that of CPDS in vitro (Figure 3a). Further investigation into the mechanism of CPDS's hemostatic effect revealed that all three CPDSs had the highest erythrocyte and platelet adhesion (*p* < 0.01) (Figure 3b–d), which is linked to the chitosan's cationic properties and macroporous structure.^[^ ^10^, ^19^ ^]^ Interestingly, the commercial chitosan sponge (CS) and CPDS groups exhibited more deformed erythrocytes with many protrusions, and fibrin‐formed fibrous junctions were observed in the CPDS group (Figure 3d). These are all hallmarks of coagulation,^[^ ^20^ ^]^ although it was anticoagulated blood with sodium citrate that used in the experiment. These findings fully demonstrated the hemostatic effect of CPDS. Similarly, CPDS also exhibits superior platelet adhesion capability than gauze, gelatin sponge, and commercial chitosan sponge (*p* < 0.01) (Figure 3e). We speculate that this is due to the macroporous nature of the CPDS, which facilitates blood absorption, allowing platelets to penetrate the material. In contrast, the commercial chitosan sponge is relatively dense, limiting contact between blood and the material. These results emphasize the impact of material structure on hemostatic performance.

To mimic a clinical resection wound of liver cancer, we utilized rat liver punching and partial liver resection models to investigate the hemostatic efficacy of CPDS (Figure 3f). In the previous research, we found that the liver bleeds quite slowly, rendering a pre‐compressed sponge ineffective for rapid hemostasis as it takes a long time to absorb enough blood and rebound fully.^[^ ^21^ ^]^ Therefore, although this strategy is adopted by most incompressible hemorrhagic materials,^[^ ^10^ ^]^ it is not applicable in the field of the liver. Hence a sponge with an 8 mm diameter was directly stuffed into the 6 mm wound. This approach facilitated rapid initial hemostasis, and the sponge exhibited good compressibility as well as plasticity, enabling it to easily adapt to any wound despite its larger diameter (Figure 3f; Figure S6, Supporting Information). Our strategy resulted in a hemostasis time of only 48.0 ± 10.0 s and a bleeding loss of 0.33 ± 0.05 g, which outperformed both gelatin sponge and commercial chitosan sponge (*p* < 0.05). In comparison, the gelatin sponge with an 8 mm diameter shrinks after absorbing blood, resulting in a hemostasis time of 110.8 ± 10.1 s and a bleeding loss of 0.63 ± 0.15 g. In addition, although the chitosan sponge has an excellent performance in the whole blood coagulation experiment in vitro, it takes 93.0 ± 8.8 s for hemostasis and the bleeding loss reaches 0.62 ± 0.22 g due to its shortcomings such as less blood absorption and inability to tightly fit the wound when used in vivo.

Hepatic lobectomy is the preferred treatment for liver cancer.^[^ ^22^ ^]^ Therefore, we established a rat model of partial liver resection for testing (Figure 3g; Figure S7, Supporting Information). Our findings indicate that CPDS outperforms gelatin sponge and commercial CS (*p* < 0.05) (Figure 3g; Figure S7, Supporting Information). The CPDS, with its soft texture, adheres to the wound instantly through the surface tension of the blood. Conversely, the stiff commercial CS fails to optimize the hemostatic effect of chitosan, and large wounds are still bleeding freely (Figure 3g; Figure S7, Supporting Information). Similarly, only a small amount of blood penetrates the gelatin sponge, weakening its hemostatic effect. It took 318.4 ± 47.5 s to stop bleeding, and the bleeding loss reached 1.85 ± 0.29 g. In contrast, CPDS takes only 183.0 ± 16.4 s, and the bleeding loss is reduced to 0.99 ± 0.15 g. Finally, hemolysis tests and coculture experiments showed that CPDSs present hemolysis rates of less than 5% and great cytocompatibility (Figure S8, Supporting Information). Technically, an adhesive coating can be added on the surface of CPDS to achieve the purpose of sealing the wound and to further improve the hemostatic effect of CPDS.

### 2.3 In Vitro Photothermal Properties and Tumor‐Killing Effect of CPDS

**Figure 4:**
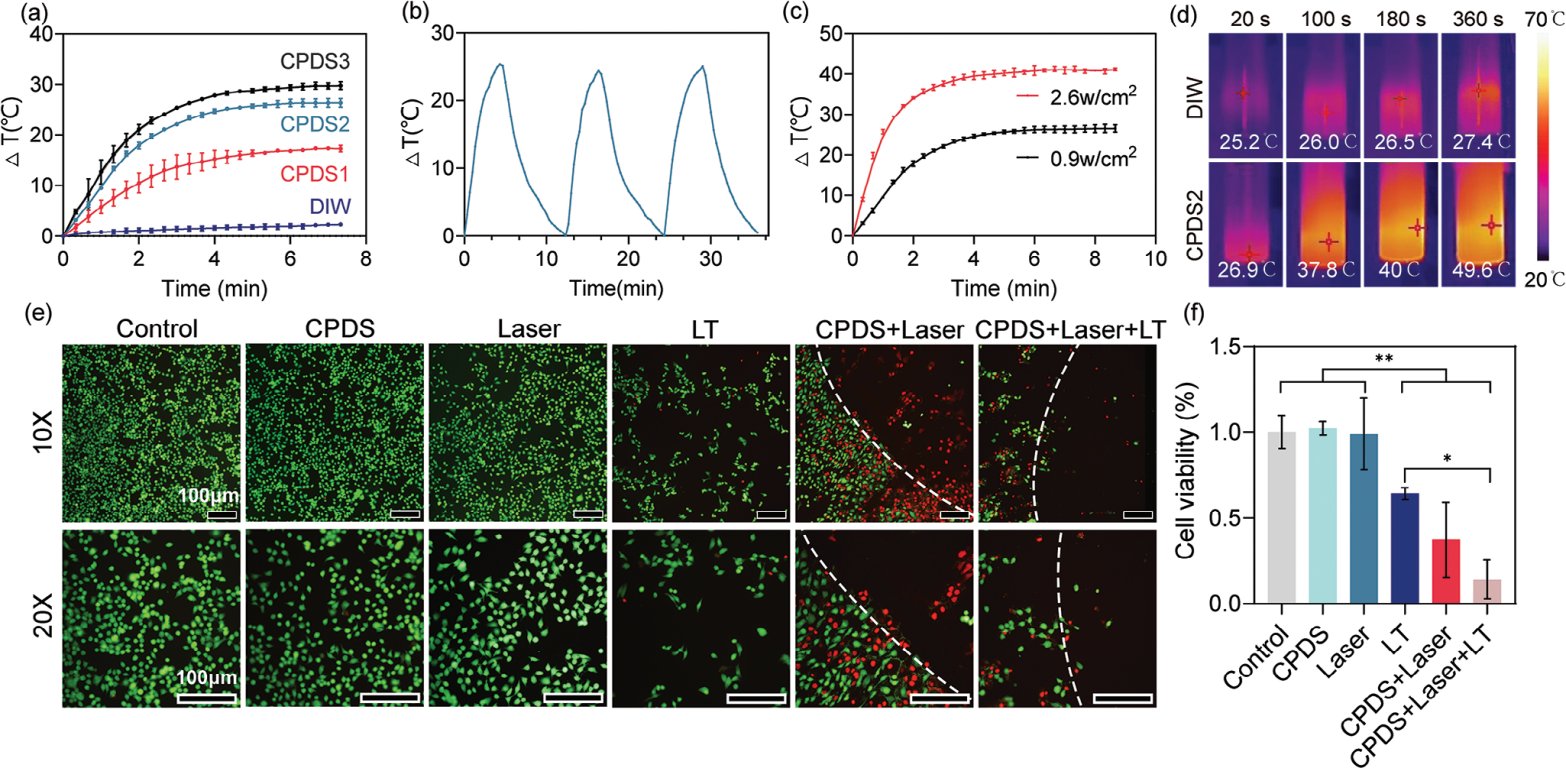
CPDSs have good photothermal conversion efficiency and tumor‐killing effect in vitro. ^* *^ *p* < 0.001, ^* *^ *p* < 0.01, ^*^ *p* < 0.05. a) ΔT–NIR irradiation time curves of the CPDSs with a light intensity of 0.9 W cm^−2^. b) ΔT–NIR irradiation time curves CPDS under NIR irradiation (0.9 W cm^−2^) after three irradiation‐cooling cycles. c) Photothermal effects of CPDS with light intensity varying from 0.9 to 2.6 W cm^−2^, respectively. d) Representative thermal images of CPDS and DIW under NIR irradiation (0.9 W cm^−2^). e) Cell morphology and f) Cell viability after photothermal treatment (0.9 W cm^−2^, 6 min) and other treatments. Live cells are shown in green and dead cells are shown in red. The right side of the dotted line is where the CPDS is placed. Scale bar: 100 µm.

Under the light intensity of 0.9 w cm^−2^, with the increase of dopamine content, the photothermal ability of the CPDS was enhanced (**Figure** **4**a). Both CPDS2 and CPDS3 can heat more than 20° in 10 min, and increasing the power further extended the temperature range, while deionized water (DIW) rose just 2.3 °C (Figure 4a–d). Notably, the CPDS's photothermal performance remained stable after repeated heating and cooling (Figure 4b). Conventional PTT typically raises the temperature above 50 °C, which can harm healthy tissue. However, since tumor cells are more susceptible to heat than normal cells,^[^ ^23^, ^24^ ^]^ mild PTT at ≈45 °C can be used to avoid such side effects. At this temperature, tumor cells undergo immunogenic death instead of cell necrosis caused by high temperature, which is conducive to the activation of tumor immunity.^[^ ^25^, ^26^, ^27^ ^]^

Based on this, we verified the anti‐tumor effect of CPDS in vitro by using a light intensity of 0.9 w cm^−2^ for 6 min (Figure 4), and the first‐line anti‐liver cancer drug lenvatinib was used as a positive control. Since the main anti‐tumor mechanism of lenvatinib is to inhibit angiogenesis, the killing rate of lenvatinib on hepatocytes in vitro was only 36% (Figure 4e,f). Furthermore, pure sponge and laser irradiation were found to have no discernible effect on tumor cells. Only when the light was converted into heat energy by the CPDS, did the tumor cells exhibit a 62% decrease in viability, surpassing that of the LT group (*p* < 0.05) (Figure 4f). When lenvatinib was combined with PTT (CPDS + Laser), it showed the best antitumor effect among all groups. This is noteworthy, as LT functions as an antiangiogenic drug and operates through a completely different mechanism than PTT. A recent study by Du et al. demonstrates that the combination of the two methods can achieve superior anti‐tumor effects.^[^^]^ Moreover, in vitro immunofluorescence assays provided more intuitive results (Figure 4e). The tumor cells in the LT group decreased uniformly across the entire field of view, while the CPDS + Laser group showed a circle of death around CPDS. Consistently, the best effect was observed with combination therapy. In vitro experiments did not reveal an obvious influence on the hemostatic effect of different CPDSs. So based on the photothermal effect and mechanical properties of the material, we selected CPDS2 for subsequent experiments.

### 2.4 Recurrence Prevention After Ectopic Hepatocellular Carcinoma Resection

**Figure 5:**
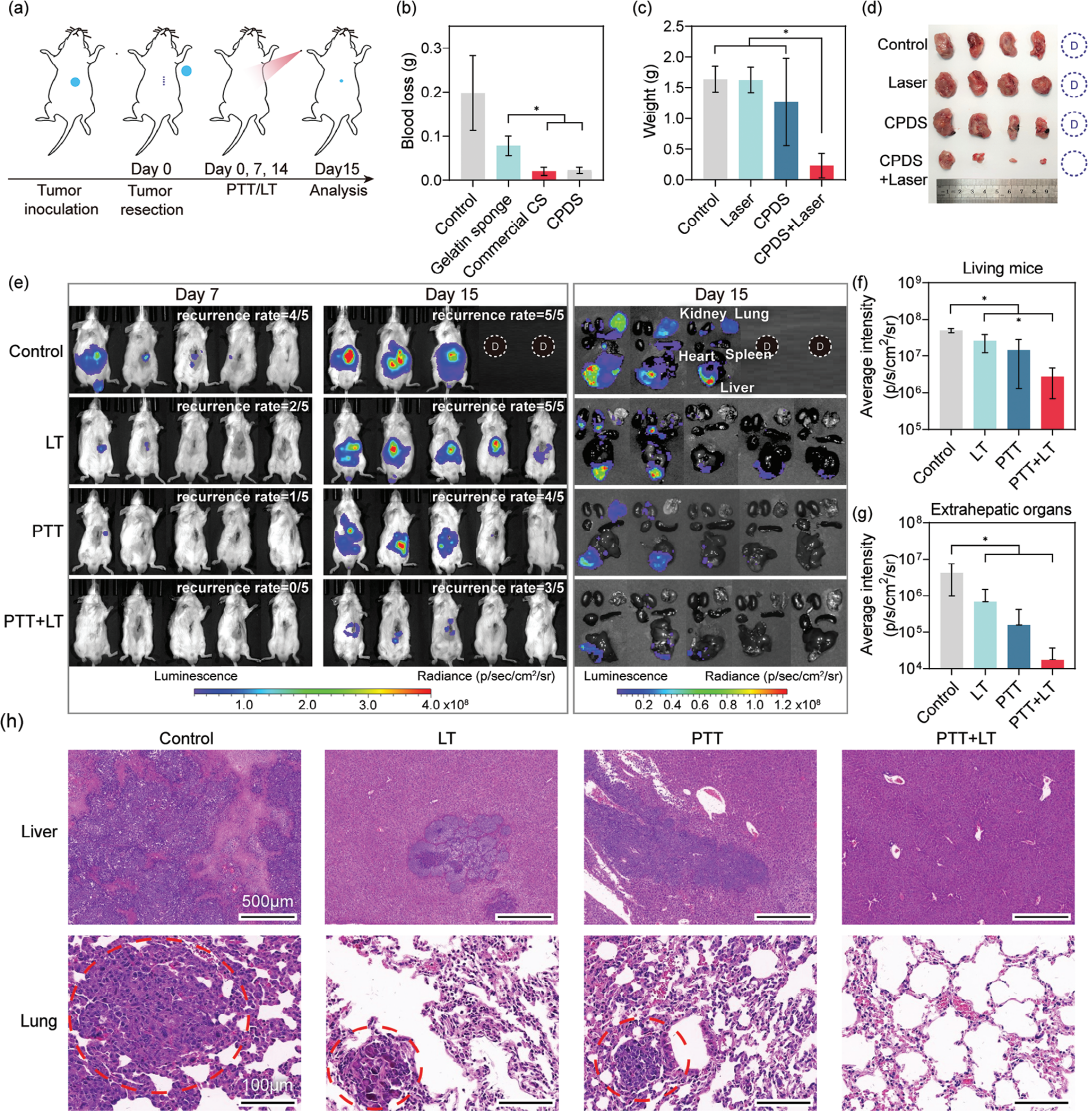
CPDS‐based PTT effectively suppressed recurrence after hepatocellular carcinoma resection and better efficacy was achieved when combined with lenvatinib. ^* *^ *p* < 0.001, ^* *^ *p* < 0.01, ^*^ *p* < 0.05. a) Schematic diagram of hepatocellular carcinoma resection model establishment and interventions. b) Auxiliary hemostatic effect of CPDS. c) Weight and d) photos of recurrent tumors 15 d after surgery in the subcutaneous HCC model (D: dead; open circle: no recurrence) (*n* = 5). e) Photos of the small‐animal living imaging at day seven and day 15 in the orthotopic HCC model. Fluorescence intensity analysis of f) living mice and of g) extrahepatic organs (heart, spleen, lung, and kidney) at day 15 (*n* = 5). h) H&E staining of liver and lungs. The darker stained part is cancer tissue, which is characterized by enlarged and darkly stained nuclei. Cancerous tissue in the lung is circled in red.

The subcutaneous and orthotopic HCC model was established in previous studies^[^ ^29^ ^]^ to evaluate the potential of preventing recurrence. On day 0, the tumor was excised (**Figure** **5**a; Figures S9a and S10a, Supporting Information). Due to the abundant blood vessels of the hepatoma, bleeding was easy to occur during the resection process. Different materials were employed to evaluate the hemostatic efficacy following tumor removal. In the subcutaneous model, CPDS achieved similar effects to commercial CS and was more effective in hemostasis than gelatin sponges (*p* < 0.05) (Figure 5b). After hemostasis, the mice were treated with different interventions. The temperature of the CPDS and the surrounding tissue can quickly rise above 45 °C under the power density of 0.9 w cm^−2^ (Figures S9b and S10b, Supporting Information). The subcutaneous model demonstrated significant suppression of tumor recurrence through CPDS‐based PTT (CPDS + Laser group). Tumor volume and weight were notably lower in this group compared to the other three groups (Figure 5c,d; Figure S9c). TUNEL staining quantifies the extent of necrosis within the tumor tissue, we observed an area of necrosis of CPDS + Laser that was more than twice as large as others (Figure S9d,e, Supporting Information). Beyond its direct thermal ablation effects, PTT also demonstrated the capacity to stimulate immune activation. Notably, the CPDS+Laser group exhibited a significant increase in mature DCs within the spleen of mice, reaching an impressive 43.03 ± 4.14%, which was substantially higher than the other groups (Figure S9f,g, Supporting Information).

Furthermore, we assessed whether CPDS‐based PTT could inhibit the recurrence of liver cancer in an orthotopic HCC model, and whether combining lenvatinib with PTT could yield superior outcomes. Lenvatinib, an angiogenesis inhibitor, has been recommended in some studies as postoperative adjuvant therapy for high‐risk liver cancer patients.^[^ ^30^, ^31^ ^]^ Figure 5e and Figure S10c (Supporting Information) reveal that mice, which received only hepatectomy, experienced relapses within 15 days of observation. CPDS‐based PTT reduced the relapse rate by 20%, and by 40% in mice receiving PTT + LT during the observation period. Interestingly, all recurrence has appeared in the liver and is preferred near the resection margin. Liver cancer tends to recur in situ, possibly due to surgical injury inducing overexpression of factors like IL‐11 that promote liver cell proliferation^[^ ^6^ ^]^ and the presence of occult lesions around the incision.^[^ ^4^, ^5^ ^]^ Thus the direct thermal effect of CPDS‐PTT effectively inhibits in‐situ recurrence by eliminating hidden lesions.

For the analysis of extrahepatic metastases, severe systemic metastasis occurred in the Control group, manifested as increased cancerous ascites (Figure 5e), intestinal metastasis (Figure S10d, Supporting Information) and severe lung metastases (Figure 5e–h), whereas no lung metastases were observed in the PTT + LT group during the observation period and only one case of kidney metastasis was identified, with fluorescence intensity of PTT + LT in extrahepatic organs significantly lower than that observed in the Control and LT groups (Figure 5g). Yet it is worth noting that while 60% of mice in the PTT group did not exhibit extrahepatic metastasis, one mouse did develop severe lung metastasis. This highlights that simple PTT may primarily eliminate intrahepatic lesions, particularly those concealed around the incision. In cases where liver cancer has metastasized to other liver lobes or distant locations, the efficacy of simple PTT will be weakened. Hence, the combined use of PTT and LT can substantially reduce the likelihood of liver cancer recurrence. The results of H&E staining are consistent with in vivo imaging, a substantial presence of metastatic tumor cells in the livers and lungs within the Control. In contrast, the PTT and LT exhibited scattered tumor cells in the livers and lungs, while it's hard to find tumor cells in the lungs from the PTT + LT group (Figure 5h; Figure S11, Supporting Information).

Additionally, flow cytometry analysis of the spleen was conducted to observe the immune‐stimulating effects of PTT. In alignment with the results from the subcutaneous HCC model, an increase in the proportion of mature DCs was observed in mice that underwent PTT, reaching 39.27 ± 5.38% in the PTT group and 36.13 ± 2.22% in the PTT + LT group (Figure S10e,f, Supporting Information). In addition, no significant differences in mouse body weight were observed (Figure S12a, Supporting Information), and serum liver function analysis indicated that both PTT and PTT+LT were at normal levels except for a slight increase in aspartate aminotransferase (AST) in some mice (Figure S12b–e and Table S2, Supporting Information). H&E staining analysis of the heart, liver, spleen, lungs, and kidneys showed that no organ toxicity was found when PTT and LT were combined (Figure S11, Supporting Information). For degradation analysis of CPDS, H&E staining demonstrated that CPDS was gradually replaced by tissue, and degraded by more than 40% within 54 days in the lysozyme environment (Figure S13, Supporting Information), suggesting the CPDS is biodegradable. This study highlights the potential of CPDS based PTT as an adjunctive therapy to prevent tumor recurrence.

### 2.5 The Combination of Lenvatinib and Photothermal Therapy Enhanced the Antitumor Effect

**Figure 6:**
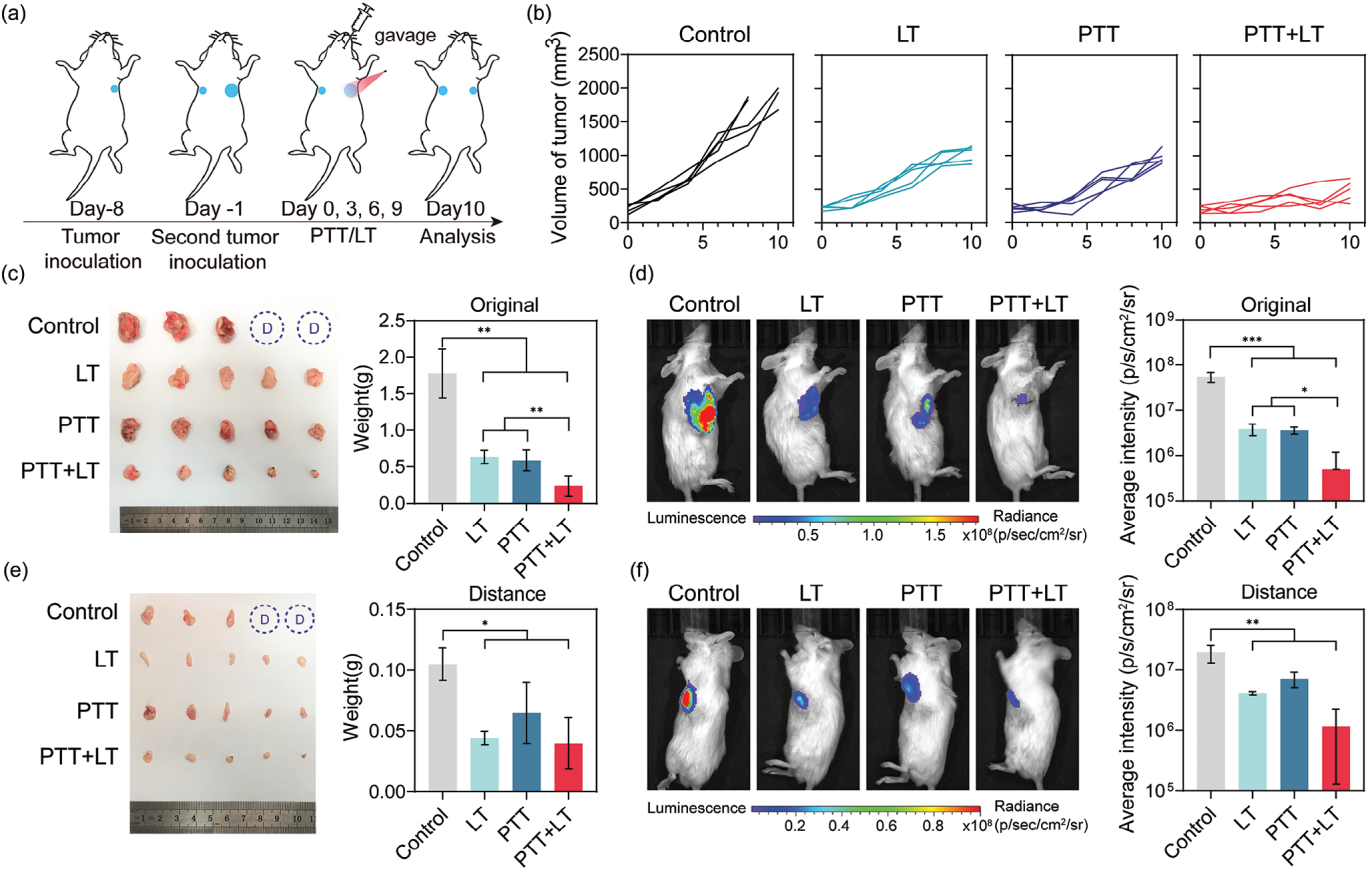
The combination of lenvatinib and PTT based on CPDS enhances the anti‐tumor effect. ^* *^ *p* < 0.001, ^* *^ *p* < 0.01, ^*^ *p* < 0.05. a) Schematic diagram of subcutaneous distant metastasis model establishment and intervention (*n* = 5). b) The original tumor volume growth curve of each mouse. c) Photos and weight of original tumor at day 10. d) The small‐animal living imaging representative photographs of the original tumor at day 10 and fluorescence intensity analysis. e) The weight and photographs of the distant tumor on day 10. f) The small‐animal living imaging representative photographs of distant tumor at day 10 and fluorescence intensity analysis.

To remove the influence of surgical procedures, we established a distant tumor model^[^ ^26^ ^]^ as shown in **Figure** **6**a, and explored the underlying mechanisms. Lenvatinib was used as a positive control. No significant differences in body weight were observed between groups (Figure S14, Supporting Information). In the blank group, the tumors grew rapidly, with the original tumor reaching a size of 1.78 ± 0.33 g on the 10th day (Figure 6b,c). Both LT and PTT (CPDS + Laser) were effective in inhibiting the growth of the original tumor, and the combination of LT and PTT (CPDS + Laser + LT) showed the best antitumor effect (Figure 6b,c). The fluorescence intensity of the tumor was 100‐fold higher in the control group than in the combined treatment group (Figure 6d), as it has been pointed out that the combination of vascular blocking therapy and PTT/PDT is a new treatment strategy to achieve all‐round attack from the outside to the inside.^[^ ^32^, ^33^ ^]^

**Figure 7:**
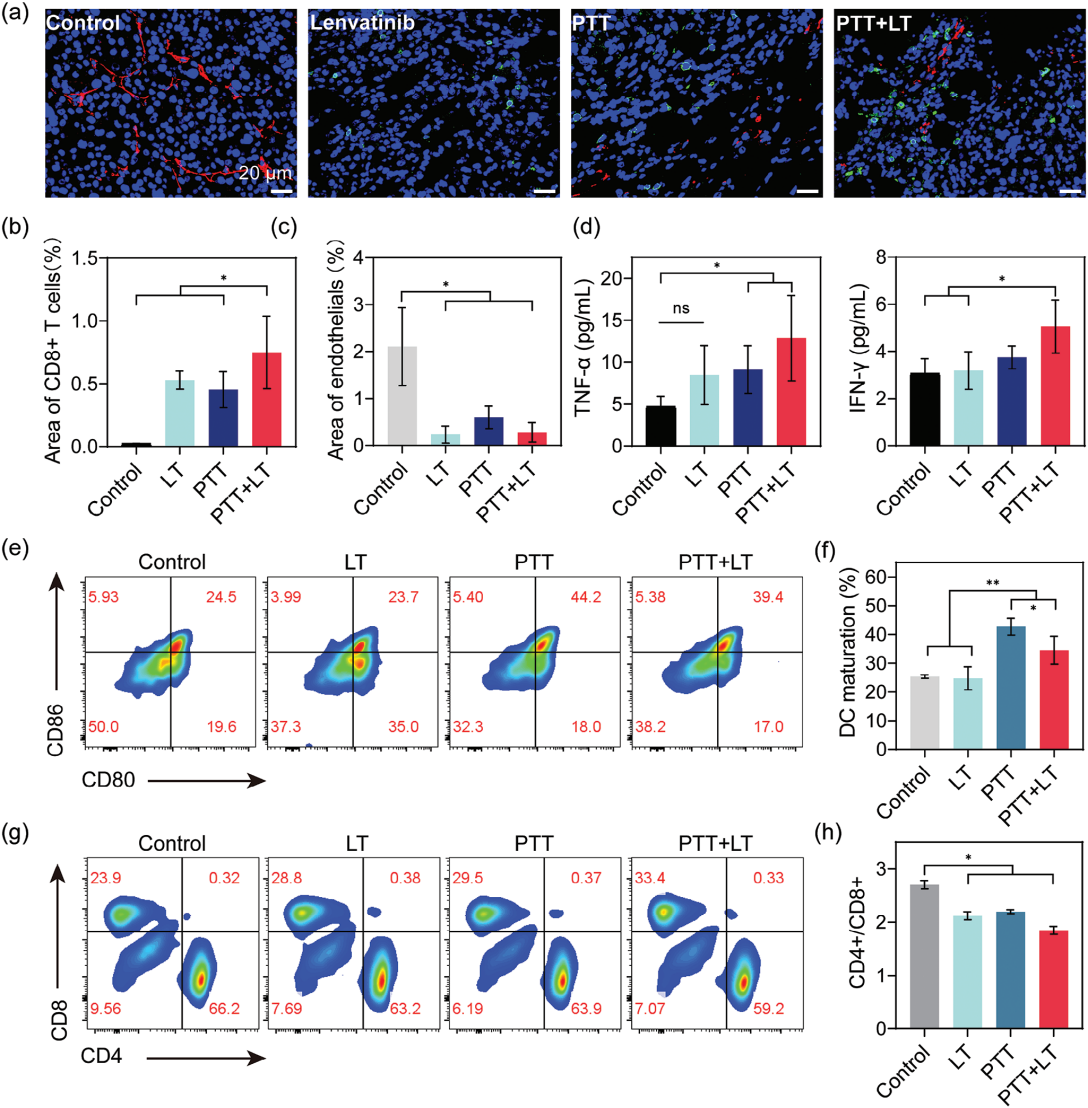
CPDSs suppress tumor growth by inducing ICD death and activating immunity. ^* *^ *p* < 0.001, ^* *^ *p* < 0.01, ^*^ *p* < 0.05. a) Immunofluorescent staining of the original tumor. Red marks CD31 +  endothelial, and green marks CD8+ T cells (CTLs). Scale bar: 20 µm. b) The area of CTLs and c) endothelial analysis. d) Plasma cytokines levels in mice receiving different treatments. e–h) Representative pictures and the relative quantification of mouse spleen flow cytometry analysis. e,f) CD80 + CD86 + indicates mature DCs. g,h) CD4‐CD8+ indicates CTLs.

Interestingly, the volume of distant tumors in the PTT group was also smaller than that in the control group (Figure 6e,f), even though we did not perform any local treatment on the distant tumors. This indicated that PTT activated immunity. To investigate this, we conducted immunofluorescence staining on tumor tissues and found that the LT, PTT, and PTT + LT groups displayed more infiltration of CD8 cells compared to the control group (Figure 7a,b). Most of the CD8 cells were located at the edge of the tumor, with the characteristics of necrosis in the center and infiltration of immune cells at the edge. Lenvatinib plays a regulatory role in the tumor immune microenvironment. C. Yi et al. pointed out that lenvatinib can also enhance the antitumor immune response of anti‐programmed cell death‐1 (anti‐PD‐1) by increasing the infiltration of CD8 + T cells.^[^ ^34^ ^]^ Our study revealed that both lenvatinib and PTT alone enhanced the marginal infiltration of tumor cells by CD8 T cells (*p* < 0.05) (Figure 7b). Moreover, the vascular density within the tumor significantly decreased in the LT, PTT, and PTT+LT groups (Figure 7a,c). Surprisingly, blood vessel density also reduced significantly in the PTT group. Studies have pointed out that heat can cause damage to vascular endothelial cells and angiodysplasia, and tumor blood vessels are more sensitive to heat damage than normal liver blood vessels, which makes them more prone to irreversible damage.^[^ ^35^ ^]^

Higher levels of cytokines TNF‐*α* and IFN‐*γ* indicating immune responses were detected in the plasma of the PTT + LT group, which were 2.7‐fold and 1.6‐fold higher than those in the control group (Figure 7d). In addition, flow cytometric analysis of the spleen more accurately confirmed immune activation throughout the mouse. Figure 7e,f and Figure S15 (Supporting Information) show that mature DC cells in groups PTT and PTT + LT increased significantly. It suggested that our treatment may have induced the ICD of tumor cells. The typical feature of ICD at the molecular level is the expression of damage‐associated molecular patterns (DAMPs), with calreticulin (CRT), heat shock proteins (HSP70 and HSP90), and high mobility group box 1 (HMGB1) taking center stage.^[^ ^36^, ^37^ ^]^ In vitro studies have shown that under CPDS‐based PTT treatment, the transcriptional expression of ANXA1, HMGB2, and HSP90 in HCC‐LM3 cells increased by more than two times compared with controls (Figure S16a, Table S1, Supporting Information). Complementary assays utilizing immunofluorescence staining brought forth a vivid upsurge in CRT expression (depicted in red) (Figure S16b, Supporting Information), an observation mirrored in the flow cytometry data, as evidenced by the discernible rightward shift of the peak (Figure S16c, Supporting Information). This is due to the transfer of CRT from the cytoplasm to the surface of apoptotic cells caused by PTT.^[^ ^38^ ^]^ CRT acts as an “eat me” signal to stimulate the antigen‐presenting function of DCs,^[^ ^36^, ^39^ ^]^ the activation of DCs further contributed to the increase of CD8+ T cells, manifested by a decreased CD4/CD8 ratio (Figure 7g,h). Ultimately, the tumor is killed by cytotoxic T cells. Thus, tumors received a double attack of external encirclement by immune cells and internal thermal apoptosis. Moreover, the combined treatment group employed lenvatinib to cut off the nutrient supply of the tumor cells, thereby producing a triple attack that obtained the optimal anti‐tumor effect.

## 3 Conclusion

In summary, we report a novel photothermal fibrous chitosan/polydopamine sponge formed by shearing‐flow‐induced fibers and polydopamine cross‐linking, with enhanced mechanical properties and dual functions of hemostatic efficacy while concurrently enhanced antitumor activity. We verified that the CPDS reduced the amount of bleeding to 0.33±0.05 g and 0.99±0.15 g in liver round defect and partial hepatectomy wound, and have investigated its mechanism, which involves promoting the adhesion of red blood cells and platelets. This rapid blood absorption is a critical first line of defense against the extravasation of tumor cells. Furthermore, excellent photothermal conversion performance that can quickly rise above 45 °C in vivo endows it with potent anti‐tumor properties, making it the second line of defense against tumor recurrence through thermal ablation of occult lesions. Remarkably, we have observed that mild PTT induces immune activation, creating an in situ “tumor vaccine” that promotes the proliferation of CTLs and infiltration in the tumor by inducing DCs’ maturation, which constitutes a third line of defense against tumor recurrence. Notably, the combination of our fibrous CPDS with lenvatinib leads to even better systemic antitumor effects. Through the combination of systemic drug application and local therapy, the fluorescence intensity of living mice in the orthotopic HCC model was reduced by 94.4% compared with the positive Control. Therefore, our comprehensive strategy represents a promising clinical intervention for adjuvant hemostasis in hepatoma resection and prevention of tumor recurrence, relying on excellent hemostatic efficacy, high tumor elimination effect, and unique immune memory protection function.

## 4 Experimental Section

### Materials

Chitosan (200–600 mPa s, degree of deacetylation ≈85%) was purchased from Tokyo Chemical Industry Co., Ltd. (TCI, Shanghai). Sodium hydroxide (NaOH), dopamine hydrochloride (DA), and sodium periodate were purchased from Shanghai Aladdin Biochemical Technology Co., Ltd. (Shanghai, China). All the reagents were used as received without further purification. Calreticulin Polyclonal for immunofluorescence is from Bioswamp (Wuhan, China), and flow cytometric analysis of calreticulin used Mouse Monoclonal Calreticulin Antibody (1G6A7) (Novus, Colorado) and Goat Anti‐Mouse IgG‐PE (Absin, Shanghai). Other antibodies for immunofluorescence and flow cytometry were purchased from Servicebio Biological Co., Ltd. (Wuhan, China) and BD Biosciences Co., Ltd. (USA), respectively. Lenvatinib was purchased from Selleck Chemicals Co., Ltd. (USA).

### Preparation of CPDS

Chitosan was dissolved in 0.05 mol L^−1^ hydrochloric acid solution to prepare a 1.2 wt.% chitosan solution. The DA was dissolved in the chitosan solution, and the mass ratios between DA and chitosan were 0.075, 0.15, and 0.225 in this work. During the homogenization (8000 rpm) with a homogenizer (IKA T25 homogenizer, Germany), the NaOH solution (1 m) was added dropwise into the system to adjust the pH to 10. After that sodium periodate (0.5:1, molar ratio) was added as an oxidant, relative to the DA under homogenization. After the oxidation of dopamine for ≈5 min, the chitosan and dopamine system formed a relatively uniform black suspension. The suspension was centrifuged at 2500 rpm min^−1^ for 10 min, and the supernatant was removed to increase the concentration. Following that, the suspension was poured into cylinder molds and frozen at −20 °C overnight. CPDSs were prepared by thawing and washing with DI water a dozen times. Subsequently, the sponges were immersed in alcohol for 12 h and then dried at ambient temperature. The sponges were coded as CPDS1, CPDS2, and CPDS3, where the original mass ratios between DA and chitosan were 0.075, 0.15, and 0.225, respectively.

### In Vivo Hemostatic Properties

All animal experiments were approved by the Laboratory Animal Committee of Wuhan University Zhongnan Hospital (the number of ethic approval: ZN2021135). 1) Standard round defect liver hemorrhage model: Cut the commercially available sponge and CPDS into cylinders with a diameter of 8 mm and a height of 3 mm. Here, a biopsy needle (6 mm in diameter and 3 mm in depth) is used to create a circular defect in the rat liver. Immediately afterward, each sample was placed into the wound, and the amount of blood loss and the duration of blood loss was assessed. 2) Partial hepatectomy wound bleeding model: Cut the commercially available sponge and CPDS into a cuboid with length and width of 3 × 1 cm. The hemorrhage model after partial hepatectomy in rats was established according to the previous research.^[^ ^7^ ^]^ Immediately after liver lobectomy, CPDS, commercial chitosan sponge, and gelatin sponge were placed at the wound site. Blood is blotted with gauze, and the amount and time of blood loss are assessed.

### Photothermal Performance

Different CPDS was cut into disks (diameter 8 mm, height 5 mm), and exposed to near‐infrared laser (MDL‐N‐980 nm–8 W, Changchun New Industrial Optoelectronics Technology Co., Ltd.). Irradiated at powers of 0.9 and 2.6 W cm^−2^, temperature changes were recorded using an infrared camera.

### In Vitro H22 Cell Killing Effect Test of CPDS

HCC–LM3 cells (1.2 × 10^6^ per well) were seeded in 24‐well plates and cultured for 24 h. 5 mg of CPDS was added to the wells. Cells were subjected to laser treatment (0.9 W cm−^2^, 6 min) in the dark. Cell viability was measured with a CCK‐8 kit. In addition, cells were stained with calcein AM/PI to observe cell apoptosis under a fluorescence microscope.

### In Vivo Anti‐Tumor Effect of CPDS

1) A subcutaneous HCC recurrence model was established according to previous studies.^[^ ^40^ ^]^ Luciferase‐expressing H22 cells (1 × 10^6^, 0.1 mL PBS) were injected subcutaneously into the axilla of Balb/c mice. After the tumor volume reached ≈150 mm^3^, all visible tumors were surgically removed and then treated respectively as follows. i) Control: Excision without treatment; ii) Laser: Laser irradiation at the wound site (0.9 W cm^−2^, 10 min); iii) CPDS: Implantation of CPDS at the resection site; iv) CPDS + Laser: Implantation of CPDS at the resection site, followed by 980 nm NIR irradiation (0.9 W cm^−2^, 10 min) on days 0, 7, and 14, and temperature changes were recorded using an infrared camera. Hemostasis was performed with CPDS, gauze, and commercial CS (same volume, all small discs 1 cm in diameter), and the blood loss during surgical resection of the tumor was recorded. The small‐animal living imaging was performed after 15 days. Heart, liver, spleen, lung, kidney, and recurrent tumors were collected for H&E staining. Part of the spleen was prepared for flow cytometry analysis.

2) An orthotopic HCC recurrence model was established according to previous studies.^[^ ^6^ ^]^ Luciferase‐expressing H22 cells (1 × 10^6^, 30 µL Matrigel) were injected into the left lobe of Balb/c mice. Two days later, the tumor was resected with an electrosurgical knife, and the resection margin was 2 mm away from the edge of the tumor.^[^ ^41^ ^]^ Half of the mice had their liver sections attached to CPDS (thickness 1 mm, length and width 0.5×1 cm, cut off the part beyond the liver if necessary). Afterward, treatment was performed as follows. i) Control: Mice were given deionized water orally every day; ii) LT: Mice were given orally lenvatinib (dissolved in deionized water, 10 mg kg^−1^day^−1^) every day; iii) PTT (CPDS + Laser): Implantation of CPDS at the resection site, followed by 980 nm NIR irradiation (0.9 W cm^−^^2^, 10 min) on days 0, 7 and 14; iv) PTT + LT (CPDS + Laser + LT): mice were orally administered lenvatinib daily based on (iii) treatment. The small‐animal living imaging was performed on day 15.^[^ ^42^ ^]^ Then mice were sacrificed and blood was collected for liver function analysis. Heart, liver, spleen, lung, kidney, and recurrent tumors were collected for H&E staining. Part of the spleen was prepared for flow cytometry analysis.

3) For the subcutaneous distant metastasis model, H22 cells were injected as previously described. The second tumor was implanted on the opposite side 7 days after the first tumor was implanted. One day later, the original side tumor received 980 nm NIR irradiation (0.9 W cm^−2^, 10 min) every 3 days, and the contralateral side tumor was not treated. The following treatment was the same as the orthotopic HCC model. The body weight and tumor growth volume of the mice were recorded. The small‐animal living imaging was performed 10 days later. Plasma and tumors were collected. The spleen was removed for flow cytometry analysis.

### Statistical Analysis

All the data represent biological replicates (*n* ≥ 3) and were shown as mean  ±  Standard Deviation (SD). Statistical differences between the two groups were determined using a *t*‐test with GraphPad Prism 8.0, while one‐way ANOVA with a Tukey post‐hoc test was applied to the comparison among multiple groups. The fluorescence data of small animal live imaging were logarithmically analyzed for statistical analysis. Statistical difference was defined by ^*^ *p* < 0.05 as significant, ^**^ *p* < 0.01 as moderately significant, and ^***^ *p* < 0.001 as highly significant, respectively.

## Conflict of Interest

The authors declare no conflict of interest.

## Author Contributions

L.M. and L.Q. contributed equally to this work and share the first authorship. C.C., Q.Y., and Z.Z. supervised the work. C.C., Q.Y., Z.Z., X.S., L.M., and L.Q. conceived the concept and designed the experiments. L.M. and L.Q. carried out most experiments and characterizations. L.M. and H.L. carried out the animal experiments. J.H. contributed to SEM characterizations. L.Q. and L.M. created the 3D illustrations. L.M. and L.Q. drafted the manuscript. C.C., Q.Y., Z.Z., X.S., L.M., and L.Q. revised the manuscript. All authors commented on the submitted version of the manuscript.

## Supporting information

Supporting Information

## Data Availability

The data that support the findings of this study are available from the corresponding author upon reasonable request.

## References

[1] M. C. S. Wong , J. Y. Jiang , W. B. Goggins , M. Y. Liang , Y. Fang , F. D. H. Fung , C. Leung , H. H. X. Wang , G. L. H. Wong , V. W. S. Wong , H. L. Y. Chan , Sci. Rep. 2017, 7.28127057

[2] M. Feng , Y. Pan , R. Kong , S. Shu , Innovation 2020, 1, 100032. 32914142 10.1016/j.xinn.2020.100032 PMC7416132

[3] W. Zhang , B. Zhang , X.‐P. Chen , Frontiers of Medicine 2021, 15, 155. 33754281 10.1007/s11684-021-0848-3

[4] J. T. Kaifi , M. Kunkel , A. Das , R. A. Harouaka , D. T. Dicker , G. Li , J. Zhu , G. A. Clawson , Z. Yang , M. F. Reed , N. J. Gusani , E. T. Kimchi , K. F. Staveley‐O'carroll , S.‐Y. Zheng , W. S. El‐Deiry , Cancer Biol. Ther. 2015, 16, 699. 25807199 10.1080/15384047.2015.1030556 PMC4622016

[5] H.‐M. Li , Z.‐H. Ye , Chin. J. Integr. Med. 2017, 23, 555. 28523536 10.1007/s11655-017-2806-0

[6] D. Wang , X. Zheng , B. Fu , Z. Nian , Y. Qian , R. Sun , Z. Tian , H. Wei , EBioMedicine 2019, 46, 119. 31375423 10.1016/j.ebiom.2019.07.058 PMC6711863

[7] Y. Cheng , Y. Gong , X. Chen , Q. Zhang , X. Zhang , Y. He , L. Pan , B. Ni , F. Yang , Y. Xu , L. Zhou , Y. Yang , W. Chen , Biomaterials 2022, 284, 121506. 35390709 10.1016/j.biomaterials.2022.121506

[8] X. Zhao , B. L. Guo , H. Wu , Y. P. Liang , P. X. Ma , Nat. Commun. 2018, 9, 2784. 30018305 10.1038/s41467-018-04998-9 PMC6050275

[9] T. Landsman , T. Touchet , S. M. Hasan , C. Smith , B. Russell , J. Rivera , D. Maitland , E. Cosgriff‐Hernandez , Acta Biomater. 2017, 47, 91. 27721009 10.1016/j.actbio.2016.10.008 PMC5508985

[10] X. Zhao , Y. P. Liang , B. L. Guo , Z. H. Yin , D. Zhu , Y. Han , Chem. Eng. J. 2021, 403, 126329.

[11] S. Y. Cao , S. Q. Wu , X. Y. Dong , M. Long , H. Lin , F. T. Liu , Y. Wu , Z. Zhao , C. J. Chen , H. B. Deng , Adv. Funct. Mater. 2023, 33, 2215059.

[12] W. W. Zeng , H. J. Zhang , Y. M. Deng , A. T. Jiang , X. Y. Bao , M. Q. Guo , Z. M. Li , M. Y. Wu , X. Y. Ji , X. W. Zeng , L. Mei , Chem. Eng. J. 2020, 389, 124494.

[13] K. F. Chu , D. E. Dupuy , Nat. Rev. Cancer 2014, 14, 199. 24561446 10.1038/nrc3672

[14] L. Qi , S. Wang , L. Chen , L. Yu , X. Guo , M. Chen , W. Ouyang , X. Shi , C. Chen , ACS Nano 2023, 17, 6317. 36944053 10.1021/acsnano.2c10462

[15] L. H Qi , C. Chen , L. Chen , L. Yu , J. Huang , S. J Wang , Z. Wang , X. W. Shi , C. J. Chen , Chem. Eng. J. 2023, 468, 143595.

[16] D. Renard , S. Tian , A. Ahmadivand , C. J. Desantis , B. D. Clark , P. Nordlander , N. J. Halas , ACS Nano 2019, 13, 3117. 30807101 10.1021/acsnano.8b08445

[17] T. Wang , Wusigale , D. Kuttappan , M. A. Amalaradjou , Y. Luo , Y. Luo , Adv. Compos. Hybrid Mater. 2021, 4, 696.

[18] F. Liu , R. Jamal , T. Abdiryim , X. Liu , Cellulose 2022, 29, 8025.

[19] H. Hattori , M. Ishihara , Biomed. Mater. 2015, 10, 015014. 25611127 10.1088/1748-6041/10/1/015014

[20] Y. Guo , Y. Wang , X. Zhao , X. Li , Q. Wang , W. Zhong , K. Mequanint , R. Zhan , M. Xing , G. Luo , Sci. Adv. 2021, 7, eabf9635. 34261653 10.1126/sciadv.abf9635 PMC8279511

[21] L. H. Qi , L. X Mu , X.J Guo , A. X Liu , C J. Chen , Q. F. Ye , Z. B. Zhong , X. W. Shi , Adv. Funct. Mater. 2023, 33, 2212231.

[22] M. Attwa , World J. Hepatol. 2015, 7, 1632. 26140083 10.4254/wjh.v7.i12.1632 PMC4483545

[23] R. S. Milleron , S. Bratton , Cell. Mol. Life Sci. 2007, 64, 2329. 17572850 10.1007/s00018-007-7135-6 PMC11138411

[24] Z. Sha , A. Goldberg , Proc. Natl. Acad. Sci. USA 2020, 117, 21588. 32817432 10.1073/pnas.2001323117 PMC7474637

[25] Y. Lv , F. Li , S. Wang , G. Lu , W. Bao , Y. Wang , Z. Tian , W. Wei , G. Ma , Sci. Adv. 2021, 7, eabd7614. 33771861 10.1126/sciadv.abd7614 PMC7997510

[26] C. He , L.D Yu , H. L. Yao , Y. Chen , Y. Q. Hao , Adv. Funct. Mater. 2021, 31, 2006214.

[27] M. Wang , M.Y Chang , P. Zheng , Q. Q Sun , G.Q Wang , J. Lin , C.X Li , Adv. Sci. 2022, 9, 2203890.

[28] Y. Du , C.L Shan , Y.C You , MJ. Chen , L.W Zhu , G.F Shu , G. Han , L. M. Wu , J. S. Ji , H. Yu , Y. Z. Du , Chem. Eng. J. 2023, 454, 140437.

[29] Y. P. Liang , M. Li , Y. Huang , B. L. Guo , Small 2021, 17, 2101356. 10.1002/smll.202101356 34382336

[30] M.‐G. Dai , S.‐Y. Liu , W.‐F. Lu , L. Liang , B. Ye , Clin Med Insights Oncol 2023, 17, 11795549231180351. 37342206 10.1177/11795549231180351 PMC10278397

[31] H. Kato , Y. Asano , M. Ito , S. Arakawa , M. Shimura , D. Koike , T. Ochi , H. Yasuoka , T. Kawai , T. Higashiguchi , H. Tani , Y. Kunimura , Y. Kondo , H. Nagata , H. Sato , A. Horiguchi , World J. Surg. Oncol. 2022, 20, 278. 36057621 10.1186/s12957-022-02740-w PMC9440518

[32] T. Zhou , X. Liang , P. Wang , Y. Hu , Y. Qi , Y. Jin , Y. Du , C. Fang , J. Tian , ACS Nano 2020, 14, 12679. 32909732 10.1021/acsnano.0c01453

[33] Z. Wei , P. Liang , J. Xie , C. Song , C. Tang , Y. Wang , X. Yin , Y. Cai , W. Han , X. Dong , Chem. Sci. 2019, 10, 2778. 30996997 10.1039/c8sc04123g PMC6419942

[34] C. Yi , L. Chen , Z. Lin , L. Liu , W. Shao , R. Zhang , J. Lin , J. Zhang , W. Zhu , H. Jia , L. Qin , L. Lu , J. Chen , Hepatology 2021, 74, 2544. 34036623 10.1002/hep.31921

[35] M. Nikfarjam , V. Muralidharan , C. Christophi , J. Surg. Res. 2005, 127, 208. 16083756 10.1016/j.jss.2005.02.009

[36] W. Li , J. Yang , L. Luo , M. Jiang , B. Qin , H. Yin , C. Zhu , X. Yuan , J. Zhang , Z. Luo , Y. Du , Q. Li , Y. Lou , Y. Qiu , J. You , Nat. Commun. 2019, 10, 3349. 31350406 10.1038/s41467-019-11269-8 PMC6659660

[37] F. Radogna , M. Diederich , Biochem. Pharmacol. 2018, 153, 12. 29438676 10.1016/j.bcp.2018.02.006

[38] E. E. Sweeney , J. Cano‐Mejia , R. Fernandes , Small 2018, 14, e1800678. 29665282 10.1002/smll.201800678

[39] L. Galluzzi , A. Buqué , O. Kepp , L. Zitvogel , G. Kroemer , Nat. Rev. Immunol. 2017, 17, 97. 27748397 10.1038/nri.2016.107

[40] Z. Zhang , G. Kuang , S. Zong , S. Liu , H. Xiao , X. Chen , D. Zhou , Y. Huang , Adv. Mater. 2018, 30. 10.1002/adma.201803217 30306650

[41] S. Liu , X. Wang , Z. Zhang , Y. Zhang , G. Zhou , Y. Huang , Z. Xie , X. Jing , Nanomed 2015, 11, 1047. 10.1016/j.nano.2015.03.001 25804412

[42] X. Yan , T. Sun , Y. Song , W. Peng , Y. Xu , G. Luo , M. Li , S. Chen , W.‐W. Fang , L. Dong , S. Xuan , T. He , B. Cao , Y. Lu , Nano Lett. 2022, 22, 2251. 35254836 10.1021/acs.nanolett.1c04413

