# Supplementary material for: Photothermal Fibrous Chitosan/Polydopamine Sponge for Intraoperative Hemostasis and Prevention of Tumor Recurrence in Hepatocellular Carcinoma Resection
Source: Adv Sci (Weinh). 2023 Nov 29;11(3):2304053. doi: 10.1002/advs.202304053 (PMC10797464; doi:10.1002/advs.202304053)
Supplement: Supplementary file 1 — Supporting Information [file ADVS-11-2304053-s001.pdf]

ADVANCED SCIENCE
Open Access

Supporting Information

for *Adv. Sci.*, DOI 10.1002/advs.202304053

Photothermal Fibrous Chitosan/Polydopamine Sponge for Intraoperative Hemostasis and Prevention of Tumor Recurrence in Hepatocellular Carcinoma Resection

Lanxin Mu, Luhe Qi, Haitao Long, Jing Huang, Zibiao Zhong, Xiaowen Shi, Chaoji Chen* and Qifa Ye**

Supporting Information

Photothermal Fibrous Chitosan/Polydopamine Sponge for Intraoperative Hemostasis and Prevention of Tumor Recurrence in Hepatocellular Carcinoma Resection

Lanxin Mu[1†], Luhe Qi[2†], Haitao Long[1], Jing Huang[2], Zibiao Zhong[1], Xiaowen Shi[2], Chaoji Chen[2]*, Qifa Ye[1]**

Characterizations: The FT-IR spectra of the raw chitosan powder and CPDS were recorded by using a NICOLET 5700 FT-IR Spectrometer (Thermo Scientific Instrument). A field emission scanning electron microscope (Zeiss Gemini SEM 500) was used to observe the morphologies of the CPDSs. The hydrophilicity of the CPDS was confirmed via a drop shape analysis system CAST 3.0 (USA KINO Industry) at room temperature. At three different positions, a water droplet of 1.0 μL was dispensed onto the surface of the sponge. The Raman spectra were measured using a confocal Raman microscope (XploRA Plus, HORIBA Jobin Yvon) with a 532 nm wavelength of the excitation laser. Raman mappings (scan range = 22 × 22 μm) were collected using the condition that the laser power was 7.0 mW, the exposure time was 0.1 s (10 Hz), the number of scans was 40, and the image pixel size was 1.0 μm. The collected spectra were preprocessed using cosmic-ray removal, noise filtering, and normalization techniques.

Mechanical measurement: The mechanical tests were performed by employing a universal testing machine (UTM6503, Shenzhen SANS, China) with a loading speed of 10 mm min^{-1}. The CPDSs were tested after being immersed in PBS solution to simulate the environment *in vivo*.

Porosity measurement: The porosity measurement was revised according to the reported method.[1] The cylindrical sponges were first weighted (W_0) before being immersed in absolute ethanol until they were equilibrated. They were weighted again (W_1) after the immersion. The volume of the sponge was calculated by measuring the length (L) and diameter (D). The porosity was calculated using the following equation:

$$\text{Porosity } (\%) = (W_1 - W_0)/(\rho \times 0.25\pi \times D^2 \times L) \times 100\% \quad (1)$$

Where ρ is the density of alcohol at room temperature.

Dynamic whole blood clotting assay: The cryogels were cut into cylinders with a mass of 25 mg. 50 μL of reprocessed whole blood solution (10 μL, 0.2 M calcium chloride) was added

separately to prewarmed cryogels (37 °C). Then 10 mL of deionized water was added at 30, 60, 90, 120, and 150s to break free red blood cells. Absorbance at 545 nm was measured. 50 μL of recalcified whole blood was added to 10 mL of deionized water, and its absorbance is the reference value (negative control). The coagulation index (BCI) is calculated as:

$$\mathrm{BCI} = [(Is - Io)/(Ir - Io)] \times 100\% \qquad (2)$$

Where *Is* is the absorbance of the sample, *Ir* is the absorbance of the reference value, and *Io* is the absorbance of the deionized water.

Blood cell adhesion test: The adhesion test of red blood cells and platelets was carried out according to previous studies. Platelet-rich plasma (PRP) was separated from sodium citrate anticoagulated blood (9:1) at 120 g. Whole blood or plasma was incubated with hemostatic materials for 30 min. All samples were then washed 3 times with DPBS solution to remove physically adhered blood cells and platelets. For the number of erythrocytes adhered, 5 mL of deionized water was poured into the sponge to rupture the erythrocytes, and the absorbance at 540 nm of the solution was measured. For the number of platelets adhered, a lactate dehydrogenase (LDH) kit (Beyotime) was used for testing. The remaining samples were fixed overnight with 2.5% glutaraldehyde and dehydrated with an ethanol gradient (50%, 60%, 70%, 80%, 90%, and 100%). Finally, the samples were dried and observed with a scanning electron microscope.

Cytotoxicity test: BRL-3A cells were inoculated in 24-well cell culture plates. 5 mg, 10 mg, and 20 mg of cryogel were placed into the wells after the cells adhered to the wall and continued to culture for 24/48/72 hours. The cytotoxicity of sponges was assessed by Cell Counting Kit-8 (CCK-8). Live/dead cell staining was additionally performed to further estimate the morphology of BRL-3A cells by confocal laser scanning microscopy.

Hemolysis test: Red blood cells were obtained by centrifuging (3000 r/min) New Zealand rabbit blood for 5 minutes. RBCs were washed with DPBS and diluted to a 5% (v/v) concentration. After the dried cryogel was pulverized, DPBS was used to form a cryogel dispersion (concentrations were 1.25, 2.5, and 5 mg/ mL). Gently mix 500 µl of cryogel dispersion and 500 µl of washed erythrocyte suspension (5% (v/v)). After incubation at 37°C for 3 h, the absorbance of the supernatant at 540 nm was measured with a microplate reader.

Degradation test: The in vitro degradation process of the cryogel was evaluated by gravimetric analysis. 20 mg (W_0) of CPDS was placed in a lysozyme solution (5 mg/mL) (dissolved in PBS), and placed in a 37°C incubator for incubation. The CPDS was taken out at fixed intervals, freeze-dried, and weighed (W_t). The lysozyme solution was changed every 2 days.The degradation rate is estimated by the following equation:

$$\text{Weight loss }(\%) = 1 - W_t/W_0 \times 100\% \quad (3)$$

In vivo degradation experiments were carried out regarding previous studies. The CPDS was placed subcutaneously or between liver lobes in Balb/c mice. Assess cryogel degradation through H&E staining.

Immunohistochemistry: Histological and immunofluorescent staining of tumor tissue. Tumor tissue necrosis was evaluated by H&E staining and TUNEL staining. For immunofluorescent staining, CD8 antibodies (green fluorescence) and CD31 antibodies (red fluorescence) show the distribution of CD8+ T cells and vascular endothelial cells in the tumor tissue, respectively. Then the fluorescence intensity of different fields of view was quantitatively analyzed using ImageJ.

Flow cytometry detection: the spleen was soaked in Hank's solution, crushed, and filtered to obtain a single-cell suspension. Specimens were depleted of red blood cells using RBC Lysis Buffer (Elabscience, China). Stain the treated samples at 4 °C using a flow cytometry antibody. All samples were treated with BD Fc Block™ to reduce non-specific staining. For dendritic cells, cells were stained with Fixable Viability Stain 780 (FVS780), anti-CD45-Alexa Fluor 700, anti-CD11b-FITC, anti-CD11c-PC7, anti-CD80-PE, anti-CD86-APC. CD45+CD11b+CD11c+CD80+CD86+ cells were defined as mature dendritic cells. For cytotoxic T lymphocytes, cells were stained with FVS780, anti-CD3-PC5.5, anti-CD4-PC7, anti-CD8-BB515 and CD3+CD4- CD8+ cells were defined as cytotoxic T lymphocytes (CTLs). After staining, analysis was performed using a flow cytometer (Beckman CytoFLEX, USA).

Measurement of cell surface CRT: Overexpression of CRT was studied using immunofluorescence and flow cytometry. In this study, three groups, namely Control, CPDS, and CPDS+Laser, underwent specific treatments: LM-3 cells in the Control group were left untreated; LM-3 cells in the CPDS group were co-cultured with CPDS (small discs with a 1 cm diameter) for 24 hours following cell adhesion; LM-3 cells in the CPDS+Laser group were co-cultured with CPDS and treated with a 980nm laser (0.9 W /cm^2, 2 minutes). After the respective treatments, the cells were further incubated for 24 hours, digested, and subsequently harvested with Mouse Monoclonal Calreticulin Antibody and Goat Anti-Mouse IgG-PE for flow cytometric analysis. For immunofluorescence analysis, cell slides were thoroughly rinsed with PBS and fixed using a 4% formaldehyde solution. Calreticulin Polyclonal was added for incubation, and the nuclei were stained using DAPI. Finally, CRT expression was quantified through fluorescence microscopy.

Quantitative real-time polymerase chain reaction (qRT-PCR): Total RNA was isolated using the RNA Extraction Kit (R4111, Magen). After reverse transcription using a reverse

transcriptase kit (Vazyme), we performed real-time PCR for ANXA1, HMGB1, and HSP90 with SYBR green (Vazyme) on Bio-Rad PCR system (Bio-Rad). The sequences of primers utilized are listed in **Table S1**. Gene expression levels are calculated by the $2^{-\Delta\Delta CT}$ method and the data were presented normalized by β-actin.

Table S1. PCR primer sequences.

**Gene**	**Forward primer(5'-3')**	**Reverse primer (5'-3')**
**HSP90**	GCTTGACCAATGACTGGGAAG	AGCTCCTCACAGTTATCCATGA
**HMGB1**	TATGGCAAAAGCGGACAAGG	CTTCGCAACATCACCAATGGA
**ANXA1**	GCGGTGAGCCCCTATCCTA	TGATGGTTGCTTCATCCACAC
**β-actin**	CATGTACGTTGCTATCCAGGC	CTCCTTAATGTCACGCACGAT

Liver function analysis: EDTA (ethylenediaminetetraacetic acid)-containing blood collection tubes was used to collect blood. After loading the sample, a fully automatic biochemistry instrument (Rayto Life and Analytical Sciences Co., Ltd., Shenzhen) was used to determine alanine aminotransferase (ALT), aspartate aminotransferase (AST), γ-glutamyl transferase (γ-GT) and albumin (ALB).

Supplementary Figures

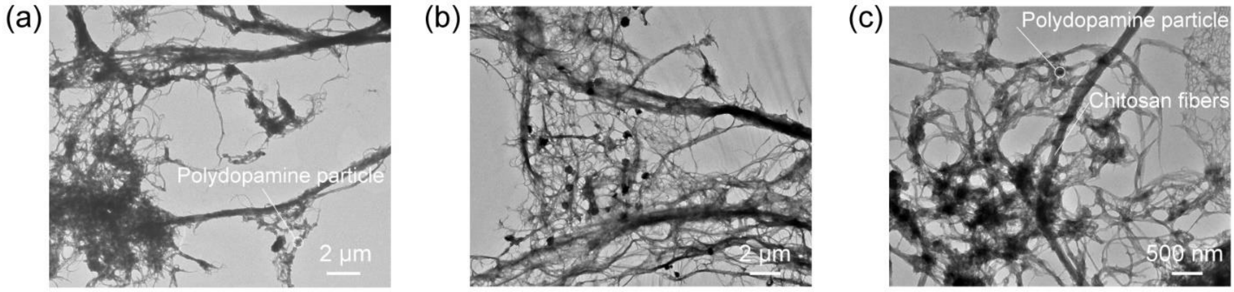

Figure S1. TEM images of the chitosan/polydopamine precursor.

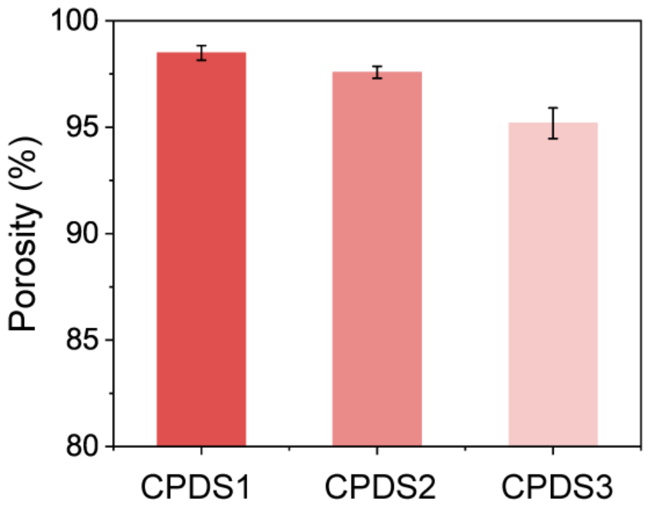

Figure S2. The porosity of the CPDSs fabricated with different polydopamine additions.

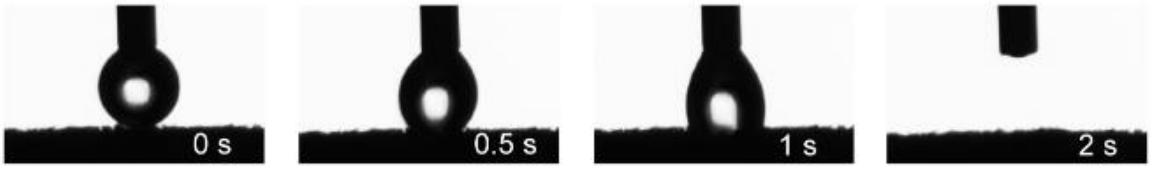

Figure S3. Water contact angles of hydrophilic surface of CPDS2 sample.

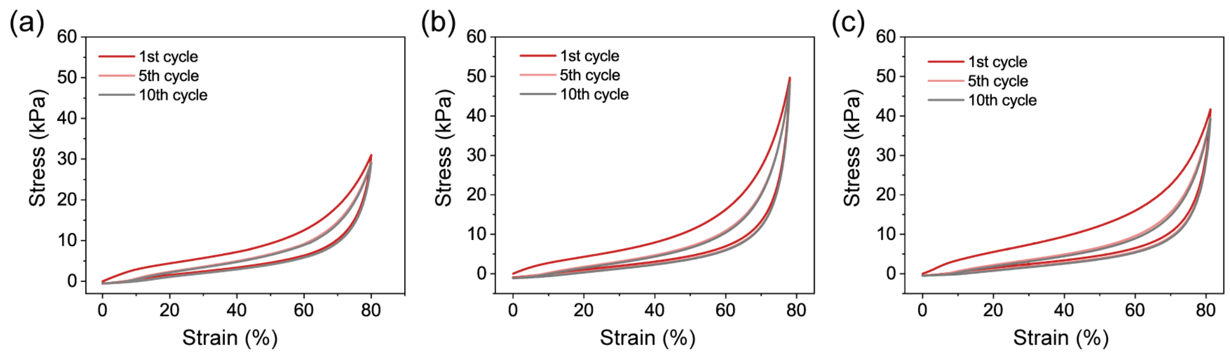

Figure S4. The cycle compression stress-strain curves of the CPDS1 (a), CPDS2 (b), CPDS3 (c) samples.

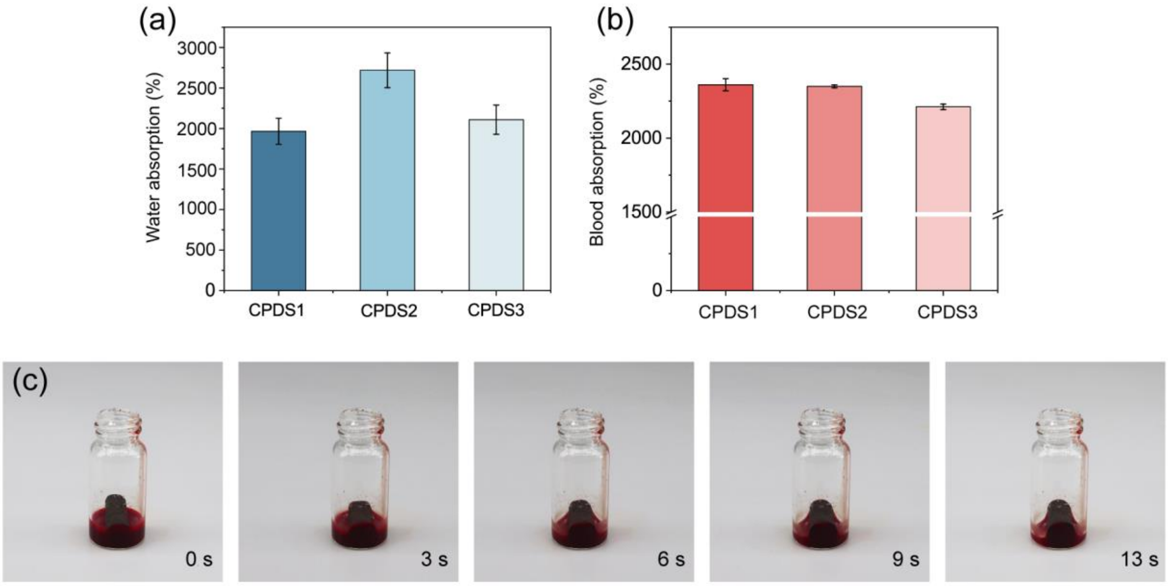

Figure S5. The water-absorbing (a) and blood-absorbing (b) properties of the CPDS at different dopamine additions. (c) The photographs of the blood absorption capability of CPDS2: absorbing 1.5 mL of blood in about 13 s.

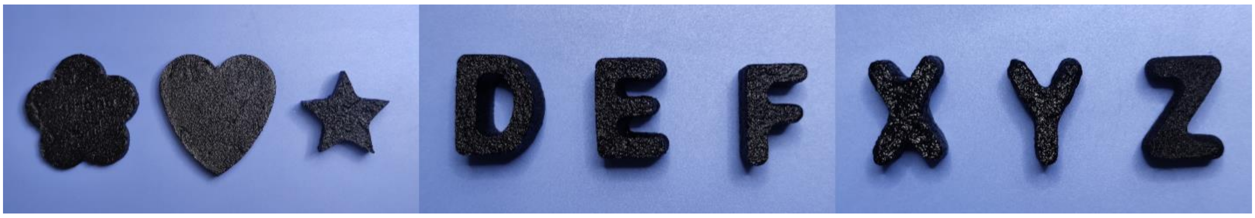

Figure S6. CPDS can be flexibly prepared into any shape.

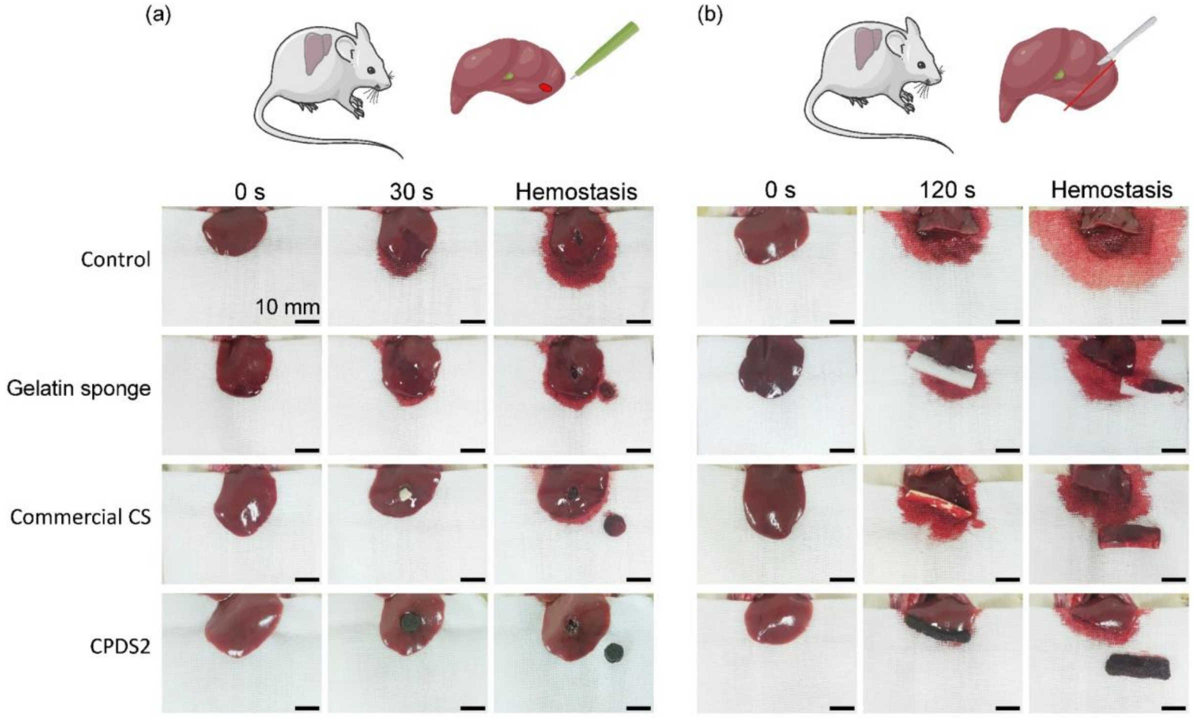

Figure S7. Photographs of the hemostasis process in the a) standard round defect liver hemorrhage model and b) partial hepatectomy wound bleeding model. Scale bar: 10 mm.

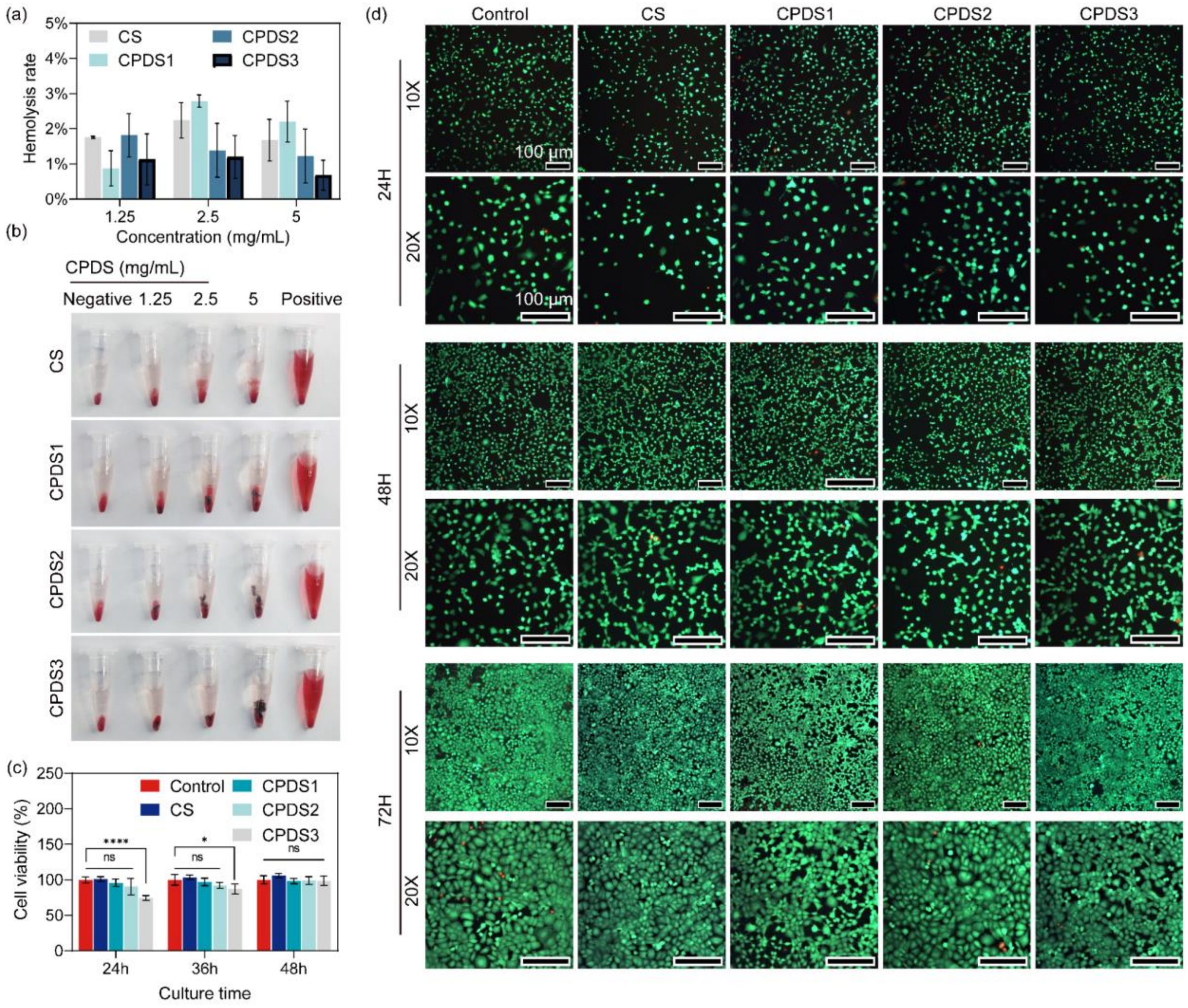

Figure S8. CPDSs have good biocompatibility and blood compatibility. * * *P < 0.001, * * P < 0.01, * P < 0.05. a) The hemolysis rate of all materials was below 5%. b) The photographs of blood compatibility experiment. c) Cell viability of CPDSs evaluated by CCK-8. d) Growth of cells co-cultured with CPDS under a fluorescence microscope. Scale bar: 100 μm.

The biocompatibility of CPDS was evaluated through hemolysis tests and co-culture experiments **(Figure S8)**. The hemolysis rate of various CPDS concentrations was less than 5%, indicating a high level of compatibility with blood **(Figure s8a, b)**. In co-culture experiments, sponges with high dopamine concentrations showed a slight loss of cytocompatibility after 24 hours of co-culture **(Figure S8c)**. This may be due to the phenol and quinone amounts on the CPDS.[2, 3] In most cases, polydopamine is usually used as a biocompatible coating in biomaterial.[4] In our experiments, the difference in cytocompatibility between the groups disappeared over time, indicating excellent biocompatibility of CPDS. Live-death staining

showed normal cell morphology and few dead cells, which again proved the good cytocompatibility of CPDS **(Figure S8d)**.

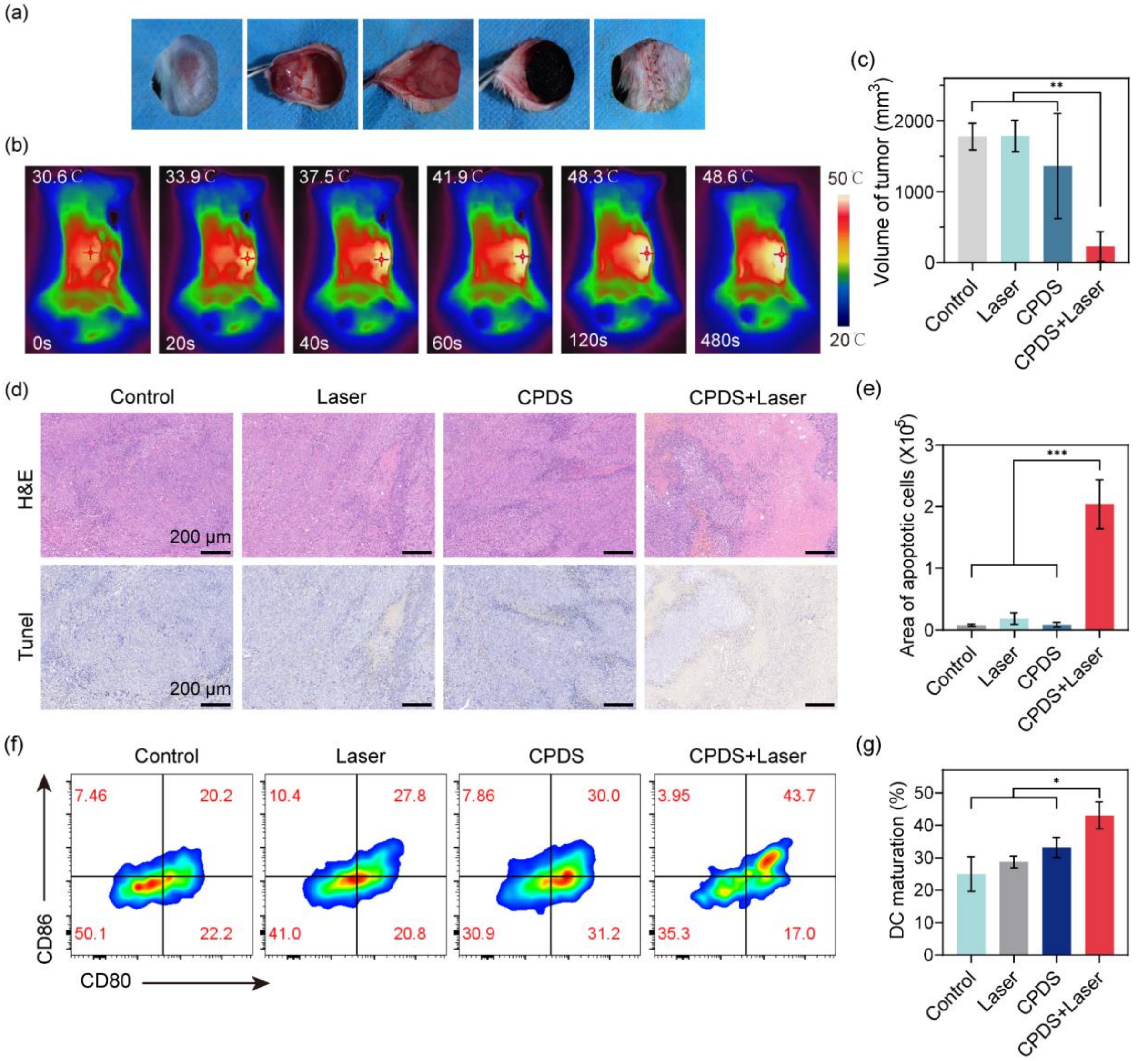

Figure S9. CPDS-based PTT used in the subcutaneous HCC model. a) Photos of tumor resection process. b) Representative thermal images of PTT based on CPDS. c) the volume of the recurrent tumor. d) Tumor tissue necrosis was shown by H&E and TUNEL staining. Scale bar: 200 μm. e) TUNEL staining positive area (brown) analysis. f) Representative pictures of mouse spleen DC flow cytometry analysis and g) Proportion of mature DCs. CD80+CD86+ indicates mature DCs.

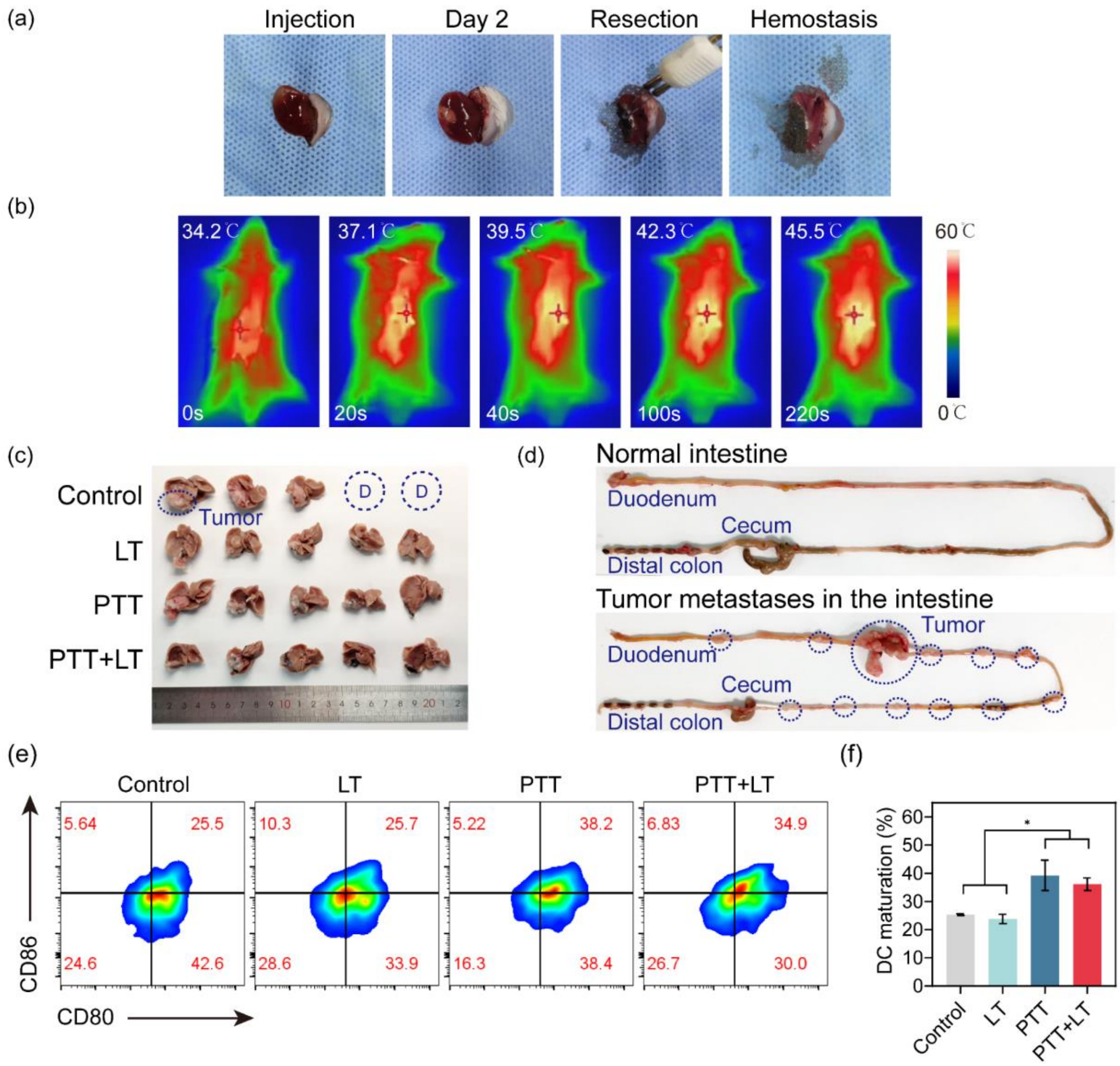

Figure S10. The combination of lenvatinib and PTT based on CPDS was used in the orthotopic HCC model. a) Photos of tumor resection process. b) Representative thermal images of PTT based on CPDS. c) Photos of recurrence tumors 15 days after surgery (D: dead). d) A large number of intraperitoneal tumor implantations were found in the Control group. e) Representative pictures of mouse spleen DC flow cytometry analysis and f) Proportion of mature DCs. CD80+CD86+ indicates mature DCs.

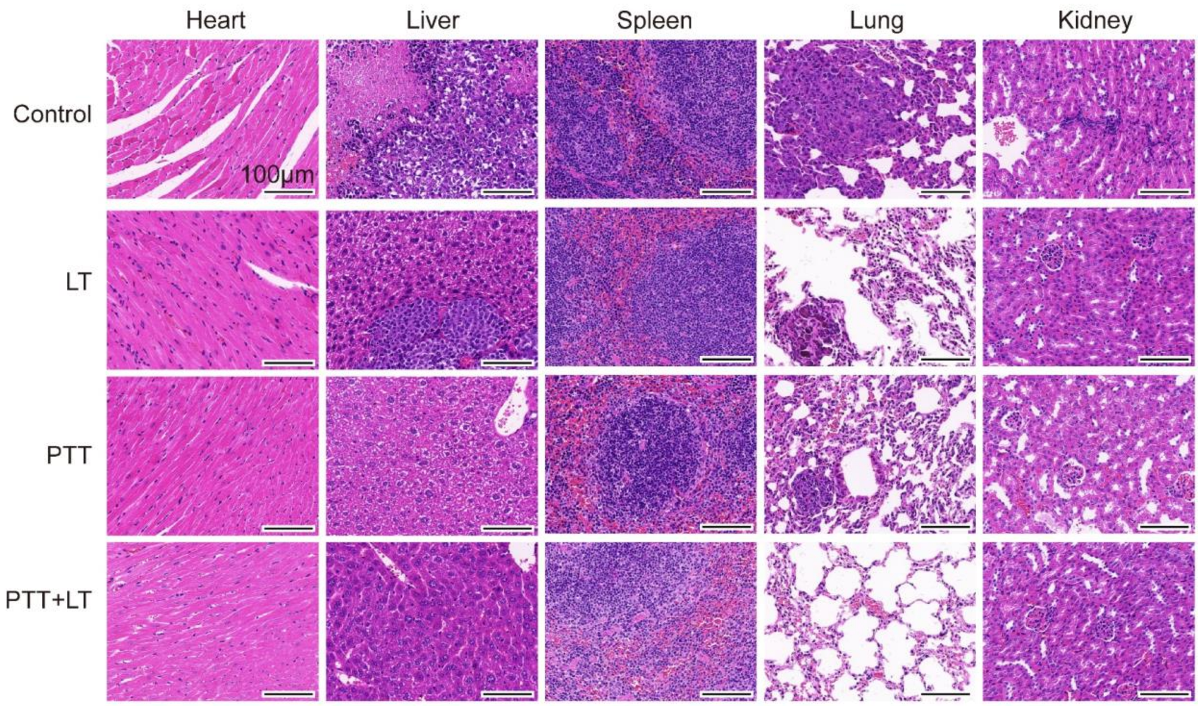

Figure S11. H&E staining showed that the heart, liver, spleen, lung and kidney of the mice in PTT+LT had normal morphology on the 15th day, and no organ toxicity was found. Scale bar: 100 μm.

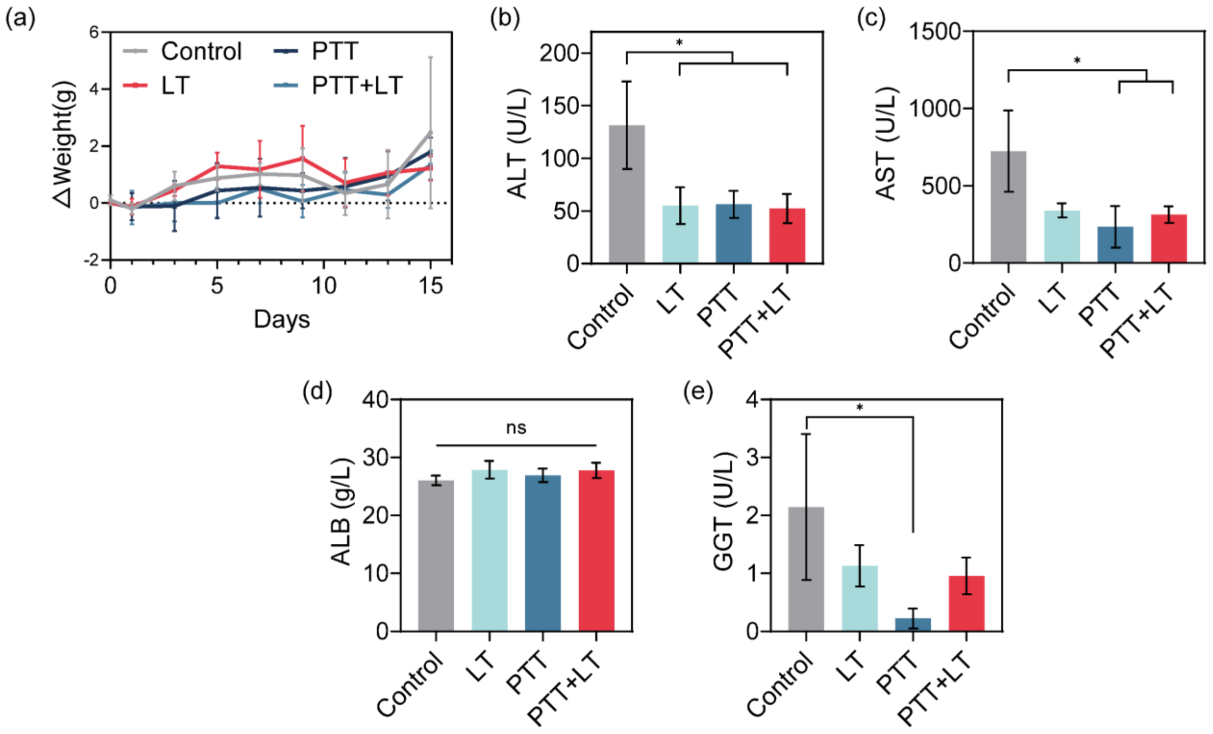

Figure S12. a) Body weight change curve of mice in orthotopic HCC model. b-e) Liver function of mice in different treatments 15 days after hepatectomy.

Mice in the Control group developed ascites as the tumors progressed, and their weight increased significantly in the final stage **(Figure S12a)** with liver function continuing to deteriorate. All mice in LT, PTT, and PTT-LT had a slight increase in AST, which may be related to the recurrence of liver cancer in some mice, the damage caused by liver cancer resection,[6] and the administration of lenvatinib.[7]

Table S2. Liver function of mice in different treatments 15 days after Hepatectomy.

	**Control**	**LT**	**PTT**	**PTT+LT**
**ALT (10.06-96.47 U/L)**	131.45±41.50	55.03±17.44*	56.32±12.93	52.32±13.88
**AST (36.31-235.48U/L)**	724.64±262.93	341.25±45.96*	235.44±134.35	314.69±53.72
**GGT (0-7.78 U/L)**	2.15±1.26	1.13±0.36	0.22±0.17	0.96±0.32
**ALB (21.22-39.15 g/L)**	26.05±0.83	27.90±1.51	26.94±1.15	27.81±1.32

* One mouse in the LT group had significantly elevated ALT and AST, reaching 2118.89 U/L and 3035.12 U/L respectively. The above is the data after excluding this mouse. Due to the possible hepatotoxicity of lenvatinib, liver function monitoring is required during treatment.

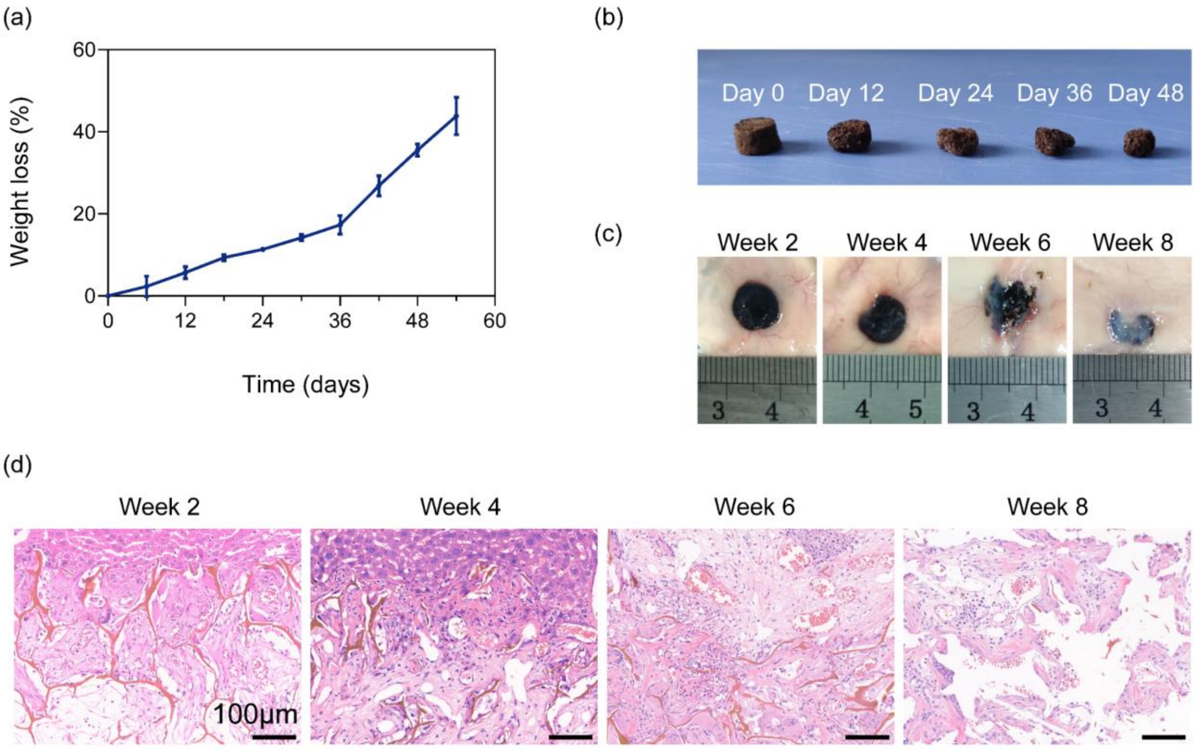

Figure S13. CPDS has good biocompatibility. a) The weight loss and b) photos of the cryogel during the in vitro lysozyme degradation test. c) Photos of subcutaneous degradation experiment. d) H&E staining showing the degradation of the material between liver lobes. Scale bar: 100 μm.

We investigated the degradation behavior of the material. In the in vitro degradation study, CPDS demonstrated significant degradation of over 40% within 54 days **(Figure S13a, b)**. As depicted in **Figure S13c, d**, the tissues in the sponge infiltrate deeper with the extension of time, accompanied by the growth of blood vessels, thus facilitating enhanced migration of cells into the material. Subcutaneous degradation experiments showed that the sponge gradually became smaller with time **(Figure S13c)**. By week 8, H&E staining exhibited a gradual disintegration of the sponge structure **(Figure S13d)**, and these data collectively illustrate the biodegradable nature of the material.

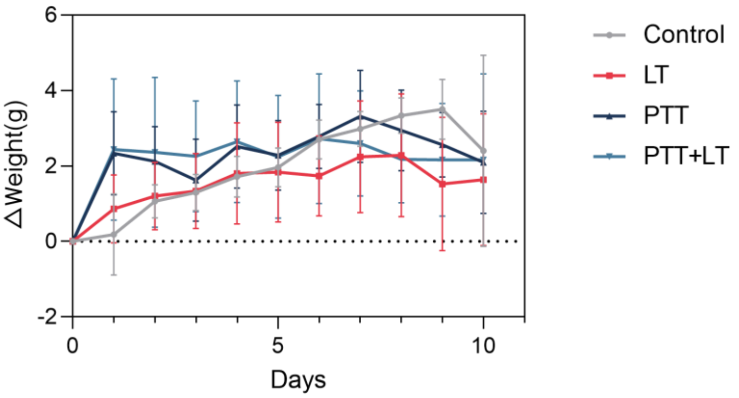

Figure S14. Body weight change curve of mice in subcutaneous distant metastasis model.

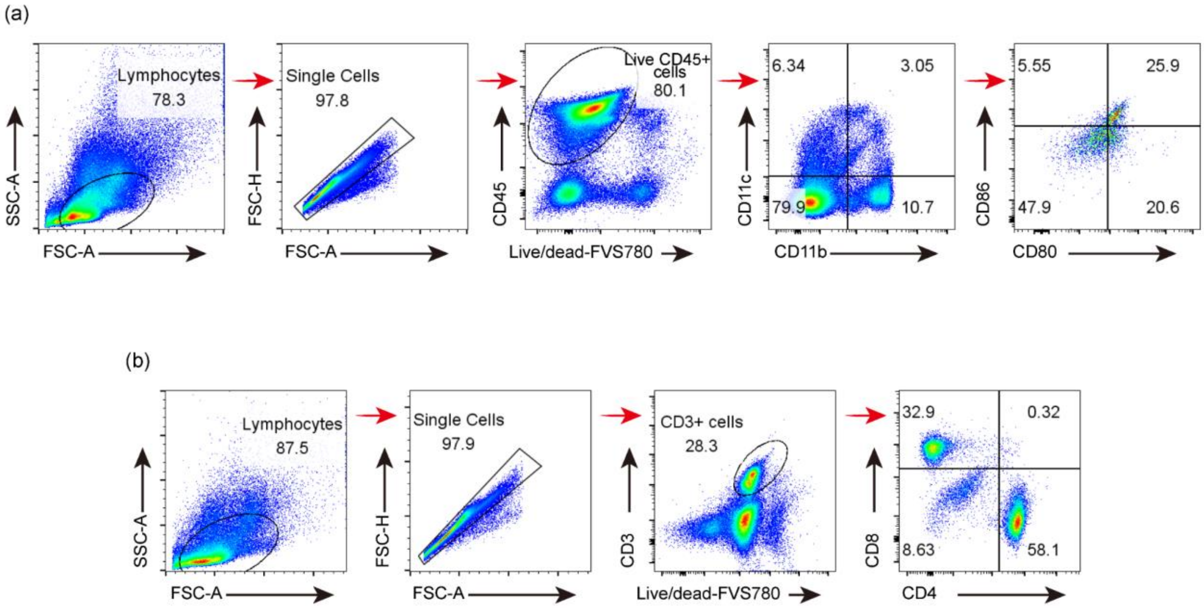

Figure S15. Gating strategy for flow cytometric analysis. a) Representative images of mature dendritic cells (CD80+CD86+). b) Representative images of CTLs (CD3+CD4-CD8+).

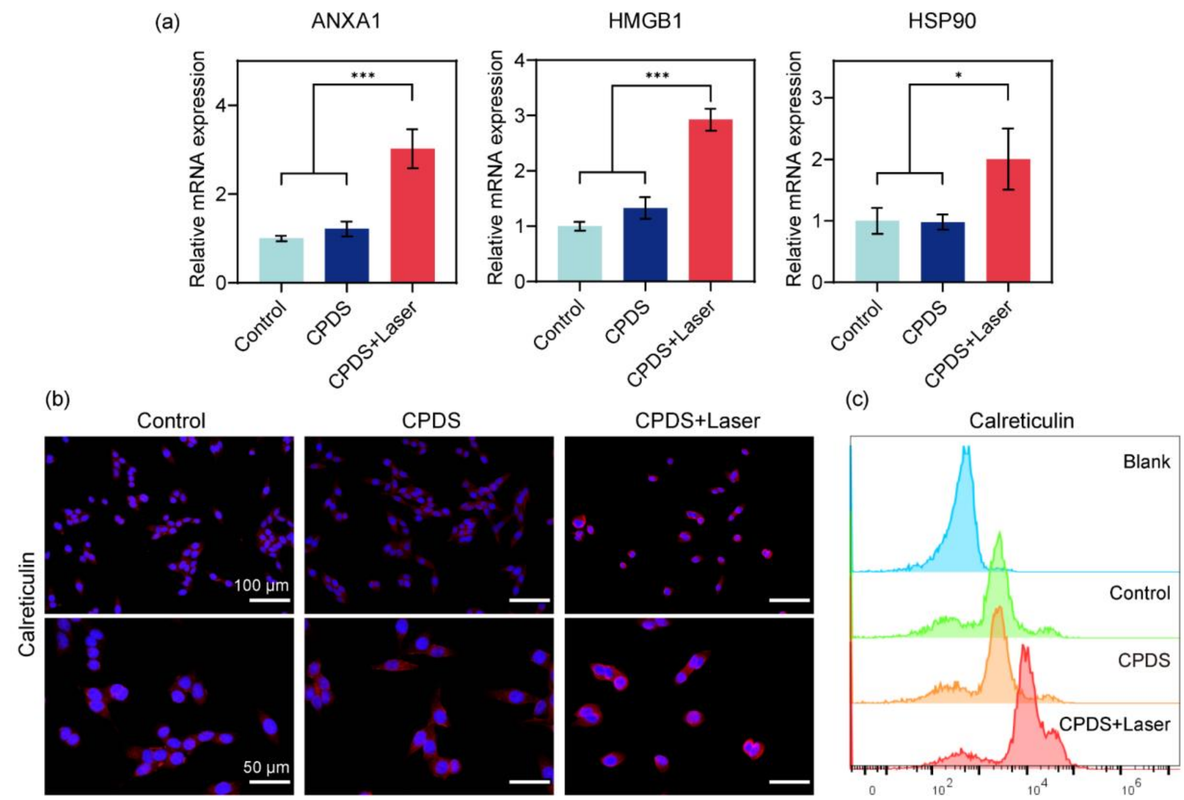

Figure S16. ICD was induced under CPDS based PTT (CPDS+Laser) in vitro. (a) The mRNA expression of ANXA1, HMGB1, and HSP90 was significantly increased in cells treated with CPDS+Laser. b, c) CRT exposure increased under CPDS+Laser, analyzed by immunofluorescence flow and cytometry, respectively.

The Blank group shows the flow cytometry analysis chart without adding fluorescent antibodies. Control performs fluorescence flow analysis on cells without any treatment. The CPDS group and CPDS+Laser group were both co-cultured with CPDS and CPDS+Laser were subjected to laser based on this basis.

Reference

[1] G. Xia, D. Zhai, Y. Sun, L. Hou, X. Guo, L. Wang, Z. Li, F. Wang, *Carbohydr. Polym.* **2020**, *227*, 115296.

[2] J. V. Uchoa Teixeira, F. R. Azevedo Maia, M. Carvalho, R. Reis, J. M. Oliveira, P. N. Lisboa-Filho, A. P. Rosifni Alves Claro, *Nanomedicine (Lond)* **2021**, *16* (1), 5.

[3] R. Luo, L. Tang, S. Zhong, Z. Yang, J. Wang, Y. Weng, Q. Tu, C. Jiang, N. Huang, *ACS Appl. Mater. Interfaces* **2013**, *5* (5), 1704.

[4] K. Park, S. Kim, Y. Jo, J. Park, I. Kim, S. Hwang, Y. Lee, S. Y. Kim, J. Seo, *Bioactive Materials* **2023**, *25*, 555.

[5] O. Oyebode, N. N. Houreld, H. Abrahamse, *Cell Biochem. Funct.* **2021**, *39* (5), 596.

[6] M. C. van de Poll, J. P. Derikx, W. A. Buurman, W. H. Peters, H. M. Roelofs, S. J. Wigmore, C. H. Dejong, *World J. Surg.* **2007**, *31* (10), 2033.

[7] S. Maruta, S. Ogasawara, Y. Ooka, M. Obu, M. Inoue, N. Itokawa, Y. Haga, A. Seki, S. Okabe, R. Azemoto, E. Itobayashi, M. Atsukawa, N. Sugiura, H. Mizumoto, K. Koroki, K. Kanayama, H. Kanzaki, K. Kobayashi, S. Kiyono, M. Nakamura, N. Kanogawa, T. Saito, T. Kondo, E. Suzuki, S. Nakamoto, A. Tawada, T. Chiba, M. Arai, T. Kanda, H. Maruyama, N. Kato, *Liver Cancer* **2020**, *9* (4), 382.
